# HER2 in Breast Cancer Stemness: A Negative Feedback Loop towards Trastuzumab Resistance

**DOI:** 10.3390/cancers9050040

**Published:** 2017-04-26

**Authors:** Babak Nami, Zhixiang Wang

**Affiliations:** Department of Medical Genetics, Faculty of Medicine and Dentistry, University of Alberta, 8-35 Medical Sciences Building, 114 St., Edmonton, AB T6G 2H7, Canada; namimoll@ualberta.ca

**Keywords:** HER2/ERBB2, breast cancer, cancer stem cell, stemness, signaling, EMT, trastuzumab, metalloproteinase, p95HER2

## Abstract

HER2 receptor tyrosine kinase that is overexpressed in approximately 20% of all breast cancers (BCs) is a poor prognosis factor and a precious target for BC therapy. Trastuzumab is approved by FDA to specifically target HER2 for treating HER2+ BC. However, about 60% of patients with HER2+ breast tumor develop de novo resistance to trastuzumab, partially due to the loss of expression of HER2 extracellular domain on their tumor cells. This is due to shedding/cleavage of HER2 by metalloproteinases (ADAMs and MMPs). HER2 shedding results in the accumulation of intracellular carboxyl-terminal HER2 (p95HER2), which is a common phenomenon in trastuzumab-resistant tumors and is suggested as a predictive marker for trastuzumab resistance. Up-regulation of the metalloproteinases is a poor prognosis factor and is commonly seen in mesenchymal-like cancer stem cells that are risen during epithelial to mesenchymal transition (EMT) of tumor cells. HER2 cleavage during EMT can explain why secondary metastatic tumors with high percentage of mesenchymal-like cancer stem cells are mostly resistant to trastuzumab but still sensitive to lapatinib. Importantly, many studies report HER2 interaction with oncogenic/stemness signaling pathways including TGF-β/Smad, Wnt/β-catenin, Notch, JAK/STAT and Hedgehog. HER2 overexpression promotes EMT and the emergence of cancer stem cell properties in BC. Increased expression and activation of metalloproteinases during EMT leads to proteolytic cleavage and shedding of HER2 receptor, which downregulates HER2 extracellular domain and eventually increases trastuzumab resistance. Here, we review the hypothesis that a negative feedback loop between HER2 and stemness signaling drives resistance of BC to trastuzumab.

## 1. Introduction

Breast adenocarcinoma arises from the epithelial compartment of the breast that consists of epithelial cells lining lobules and ducts of mammary glands. A breast tumor is a complex tissue containing cancerous cells, and various other cell types with different morphological and phenotypic characteristics, including genetics, epigenetics, gene expression, metabolism, motility and “stemness” properties. Among all subtypes of breast cancers (BCs) two common ones include invasive ductal carcinoma that starts in a milk duct of the breast and invasive lobular carcinoma that starts in the lobules. These subtypes are able to undergo metastasis. BC cells are also classified into several subtypes based on three individual hormone receptors including estrogen receptor (ER), progestin receptors (PR), and human epidermal growth factor receptor-2 (HER2). These classes include (i) basal-like tumors that are often called triple-negative (ER−, PR− and HER2−) BCs (TNBC); (ii) luminal A and B BCs that are ER+; (iii) HER2+ BCs that overexpress HER2 and are responsive to HER2 targeting adjuvant therapies and (iv) claudin-low BCs that are often triple-negative and show weak cell-cell adhesion [1]. TNBC is associated with a poor patient prognosis because of lack of the triple receptors as exquisite targets for therapeutic adjuvants.

HER2+ BCs show faster growth and greater invasiveness, but are responsive to anti-HER2 therapy. HER2 sends growth signals from cell membrane to the nucleus, therefore its overexpression is associated with poor prognosis in BC [2,3,4,5]. In the past two decades HER2 has been exploited as a potential target to treat BCs for several reasons: (i) HER2 expression levels correlate directly with BC invasion and prognosis; (ii) HER2 is a receptor tyrosine kinase with high potency to activate downstream signaling pathways involving tumor growth; (iii) HER2+ tumors exhibit significantly more HER2 receptors on the cell surface, which serves as a useful hallmark to distinguish between normal cells in pathological characterizing; (iv) The extracellular domain of HER2 provides very stable epitopes and putative targets to design and test tumor cell-targeting neoadjuvants.

Mammary tissue contains mammary stem cells that can self-renew and can differentiate into luminal and basal epithelial cell layers including ductal, alveolar and myoepithelial cells of mammary gland. Like basal-like and claudin-low subtypes BC, normal mammary stem cells are triple-negative, with active stemness signaling such as Notch and Wnt/β-catenin pathways, and with high expression of the epithelial-mesenchymal transition (EMT) elements. Thus, the mammary stem cells may be the origin of normal and cancerous breast cells [6,7]. Transforming normal breast stem cell to BC stem cells (BCSCs) with the properties of both stem and cancer cells is a major step in breast tumorigenesis. BCSCs are able to self-renew and differentiate to epithelial cancer cells with various gene regulatory networks. BCSCs definition emerged shortly after the discovery of only a small fraction of mammary tumor cells being able to form colonies or new tumors. In 2003, All-Hajj et al. [8] reported that a small fraction of mammary tumor cells with self-renewal potency and expressing certain surface markers is able to form colonies or new tumors. They found that a small fraction of cells exhibiting CD44+/CD24−/Lineage (Lin)− phenotype on surface had higher tumor-forming ability in immunocompromised mice and self-renewal property in reiterated passage than CD44+/CD24+/Lin− cells. In this experiment only cells with CD44+/CD24−/Lin− phenotype (100 such cells) were able to grow to form tumors in the animals, whereas tens of thousands of cells with other phenotypes failed to form tumors [8]. Hence, there is a positive relationship between the size of the subpopulation of BCSCs and the tumorigenesis, tumor invasion and refractory [8,9,10]. In recent years, many reports have been published focusing on BCSCs in different subtypes of breast tumors. Among them, some results suggest positive role of HER2 receptor in the emergence of BCSCs inside breast tumors. In this paper, we review the interaction of HER2 with stemness signaling pathways, which enables HER2+ BC cells to attain stem cell properties and trastuzumab resistance.

## 2. CD44, CD24 and ALDH Are the BC Stemness Markers

CD44 is a multifunctional transmembrane glycoprotein involved in binding of the cell to extracellular matrix hyaluronic acid (HA) and plays roles in cell-cell interactions, cell adhesion, growth, proliferation, survival, motility, migration, angiogenesis, and differentiation [11,12]. CD44 also interacts with many other ligands including Osteopontin, Collagens, Fibronectin, Integrin, Laminin and metalloproteinases (MPs) and is associated with many malignancies, chronic inflammatory, and autoimmune dysfunctions [13]. CD44 is encoded by a highly conserved gene located on chromosomal location 11p13 and consists of 20 exons and 19 introns [14]. CD44 gene generates more than 20 isoforms (known as “variant”) from extensive RNA alternative splicing of the ten central exons. The CD44v isoforms mediates and promotes the activation of many signal transduction pathways initiated by HER2, T cell receptor (TCR), integrin and other receptor tyrosine kinases [15]. For example, CD44/HER2 signaling increases activation of Wnt/β-catenin signaling while CD44/EGFR signaling leads to the activation of TGF-β [16,17]. It is also well-documented that the cleavage of CD44 following ligand binding induces stemness and EMT [18,19].

CD24 is a heavily glycosylated small transmembrane glycoprotein anchored in cell membrane by glycosyl-phosphotidyl-inositol. Like CD44, in cancer, CD24 is involved in cell-cell and cell-matrix junction and in cell migration, and is a significant marker for tumor prognosis as well as diagnosis. CD24 acts as a ligand for P-selectin that is an abundant protein in thrombin-activated platelets and endothelial cells [20]. Therefore, CD24 promotes cancer cell migration and metastasis by facilitating the attachment of cancer cells to activated platelets and endothelial cells [21,22]. CD24 also interacts with chemokine (C-X-C motif) receptor 4 (CXCR4) and the stromal cell-derived factor 1α (SDF1α) receptor [23]. It is a negative regulator of nuclear factor-kappa B (NF-kB) and MAPK signaling pathways in CD44-positive tumor cells [24], and a positive regulator of Sarcoma (Src) and STAT3 [25].

In 2007, Ginestier et al. [26] introduced aldehyde dehydrogenases (ALDHs) as additional markers for the BCSCs. The ALDH enzymes are a family of conserved enzymes that oxidize aldehydes, and have 19 isoforms localized to various cellular compartments including cytosol, mitochondria, endoplasmic reticulum and the nucleus [27,28,29]. ALDHs are responsible for the oxidation of aldehydes to their corresponding carboxylic acids and catalysis of retinaldehyde to retinoic acid. They also mediate the inactivation of alkylating agent cyclophosphamide analogous and other xenobiotics. ALDHs also play roles in detoxification pathways, cyclophosphamide metabolism, and biosynthesis of retinoic acid, folate, amino acid, and ethanol [27]. Stem cells from a variety of tissues show high levels of ALDH activity, which is a characteristic of “stemness”. Normal (hematopoietic and neural) and cancer stem cells are enriched in cells with high levels of ALDH activity. ALDH high cells with low side scatter are self-renewing and multipotent [30]. ALDH+ cells isolated from BC cell lines were highly invasive, exhibited high potency of self-renewal, and resistant to hypoxia and chemotherapeutic drugs when compared with ALDH− cells [31].

In breast tissue CD24 is expressed on more differentiated cells while CD44 is expressed on more progenitor-like cells. Normally, CD44+/CD24− phenotype represents undifferentiated basal/mesenchymal-like cells whereas the cells with CD44−/CD24+ phenotype are luminal/epithelial-like cells [8,32]. CD44+/CD24− BC cells are highly tumorigenic and are correlated with the presence of distant metastases compared to the cells carrying other markers [8,33,34]. The majority of tumor cells derived from metastatic sites of BC patients were highly enriched for a CD44+/CD24− subpopulation [35,36,37]. While CD44+/CD24− cells may be sensitive to some inhibitors [38,39], they often drive tumor resistance to traditional therapies [10]. Studies with tumor tissues from the BC patients who had already received chemotherapy revealed an increased percentage of mammosphere-forming cells with CD44+/CD24− phenotype subpopulation [40]. In BC cells, the gene expression profile following chemotherapy is very similar to that of CD44+/CD24− cells. This suggests that the remaining BC cells could be CD44+/CD24− cells [41]. In addition to these markers, high expression of CD133 was also found in BCSCs. BC cells with phenotypes CD44+/CD24−/ALDH+ and CD44+/CD133+/ALDH+ showed increased tumorigenicity and metastases when compared with the non-stem cancer cells [42].

## 3. HER2 Is Eminent in BC Stemness

HER2 is a proto-oncogene located in chromosomal location 17q21 and encodes a 185 kDa transmembrane glycoprotein in tissues of epithelial, mesenchymal, and neural origin [43,44,45]. HER2 belongs to the ERBB family of receptor tyrosine kinases, which comprises four members: ERBB1/HER1 (EGFR), ERBB2/HER2, ERBB3/HER3, and ERBB4/HER4. Signal transduction from these receptors is initiated by ligand binding to the extracellular domain of the receptor followed by receptor dimerization and trans-autophosphorylation of specific tyrosine residues within the cytoplasmic domain [46]. However, HER2 is an orphan receptor and is always in the active conformation [47]. Among HERs, HER2 is a preferred binding partner for heterodimerization with other HER receptors and is also homodimerized when overexpressed [46]. Homo- and hetero-dimerization of HER2 leads to the activation of several signaling pathways including PI3K/AKT and MAPK pathways and many other critical signaling elements such as STAT3 and c-JUN. These signaling pathways regulate cell division, proliferation, survival, migration, differentiation, apoptosis, and cell motility [48,49,50]. In addition, it has been reported that carboxy-terminal part of HER2 (with approximately 90–100 kDA known as p95HER2) translocates to the cell nucleus following its cleavage and act as a nuclear factor interacting in transcriptional regulation of certain genes [51,52,53].

HER2 overexpression is a poor prognostic factor in BC. It is overexpressed to >10 times more receptor per cell in 15-25% of all diagnosed BCs known as “HER2+ BCs” [2,3,4]. HER2 overexpression are mostly due to gene amplification. HER2+ BC cells grow faster and spread more aggressively when compared to HER2− breast tumors [2,3,4,5]. Meta-analysis studies reveal that about 17% of patients with early BC have HER2+ tumors while this ratio may rise to 30% of BCs in all grades. Patients with HER2+ BC live one-third shorter than the patients with a HER2− tumor, and the HER2 gene amplification is also correlated with the shorter relapsing time of the disease [54].

Some evidence suggests that HER2 may be a novel regulator of BCSCs. It is found that ALDH+ BCSC-enriched tumors were associated with HER2 overexpression [26]. Korkaya et al. [55] showed that HER2 overexpression was positively correlated with increased subpopulation of mammosphere-forming ALDH+ BCSC in BC cell lines as well as xenograft tumors. Overexpression of HER2 is also correlated with the increased expression of stem cell markers Oct3/4, Notch1, Notch2, Jagged1 and Gli1 through activation of PI3K/AKT pathway. Moreover, targeting HER2 with trastuzumab, a HER2 targeting monoclonal antibody, leads to declined ALDH+ cell subpopulation [55]. Injecting HER2 overexpressing ALDH+ cells into the mammary fat pads of NOD/SCID mice generates tumors with 4-fold more BCSCs than the tumor that developed from ALDH+ cells with normal HER2 expression. In addition, ALDH and HER2 are found co-expressed in invasive cells of luminal breast tumors [56]. Several clinical and preclinical studies demonstrate that HER2 blockade with adjuvants could reduce CD44+/CD24− and ALDH+ BCSC subpopulation inside the tumors [40,56,57,58]. A preclinical study reveals that HER2 expression in mammosphere-forming BC with high levels of the stem cell markers Oct4 and Bmi1 is 2 to 7-fold higher than other group of the cells from the same origin. This suggests that high HER2 expression is associated with BCSC properties. In parallel with HER2 expression in these cells, higher level of Notch signaling is also observed in the cells. Depletion of Notch1 leads to a significant decrease in HER2 expression in the mammosphere-forming cells. Treatment of mice bearing tumors raised from these cells with trastuzumab results in a significant regression in tumor growth. The cells derived from these tumors are unable to generate new tumors through in vitro tumor passage [59].

On the other hands, HER2 is also highly expressed in undifferentiated human embryonic stem cells (ESCs). The evidence that HER2 has pleiotropic effects on multiple cell types and organs suggests that HER2 crosstalks with variety of signaling pathways that are essential in the maintenance and/or differentiation of ESCs [60,61]. Interestingly, HER2 is suggested a positive factor in development of normal mammary gland and breast tumor by interaction with stemness signaling pathways. For example, HER2-mediated activation of PI3K/AKT signaling, leads to enrichment of ALDH+ BCSCs in BC cell culture and tumor xenografts through up-regulating Wnt/β-catenin [55].

## 4. HER2 Blockade May Target BCSCs

Lapatinib is a small molecule dual inhibitor of tyrosine kinase activity of HER2 and EGFR. It is used in combination with trastuzumab to treat advanced or metastatic HER2+ BC and is currently under phase III clinical evaluation [62,63]. Lapatinib inhibits HER2+ BC growth both in preclinical and clinical studies and improves survival rate of patients. lapatinib in combination with trastuzumab showed complementary effects of HER2 blockade and improved response in patients with HER2+ BC [64]. In addition, Lapatinib can cross the blood-brain barrier, therefore it provides an effective treatment option for patients with brain metastases [65]. Lapatinib targets cancer stem cells as well. Treatment with lapatinib inhibits mammosphere-formation of CD44+/CD24− BCSCs isolated from HER2+ BC cell lines [57]. It also decreases the percentage of ALDH+ cells by approximately 10–100-fold. Treatment with combination of lapatinib and doxorubicin increases cell death rate from 27.8% at single-agent treatment to 75.1% after combined treatment [57]. Li et al. [40] examined the post-chemotherapeutic CD44+/CD24− BCSC subpopulation in 31 BC patients with HER2− tumor who received docetaxel or doxorubicin and cyclophosphamide for 12 weeks at standard doses (group 1) and in 21 patients with locally advanced HER2+ BC who received lapatinib for 6 weeks followed by docetaxel and trastuzumab for 12 weeks at standard doses (group 2). Seven of 31 (23%) patients from group 1 showed pathological complete response for conventional chemotherapy, while the pathological complete response rate in the patients from group 2 was 62%. The percentage of CD44+/CD24− cancer stem cell in bulk tumor of group 1 group is increased from a mean of 4.7% at baseline to 13.6% (*p* < 0.001) after 12 weeks of chemotherapy. In addition, an increased mammosphere-formation efficiency (MSFE) from 13.3% at baseline to 53.2% (*p* < 0.001) is observed in the MSFE assay of tumor biopsies from these patients after conventional chemotherapy. Interestingly, the baseline CD44+/CD24− cell BCSC population in the HER2+ BCs is higher than in the HER2- tumors (10.0% versus 4.7%). This suggests that HER2 expression may be a positive factor for BCSC self-renewal. More interestingly, the post-chemotherapeutic percentage of CD44+/CD24− cancer stem cell in HER2+ tumors is reduced from 10% at baseline to under 8% after 6 weeks of lapatinib therapy [40]. These results demonstrate the role of HER2 in breast tumor invasion and chemoresistance through up-regulating BCSC inside the tumor and the hypothesis that BCSCs are mostly responsible to tumor resistance and post-therapeutic cancer relapse.

In a preclinical study, treatment with 2.5 μM lapatinib significantly inhibits mammosphere-forming ability of CD44+/CD24−/Lin− phenotype BCSCs more than 80% (*p* < 0.03) and reduces the subpopulation of the BCSCs from 16% to 3% (*p* < 0.002). In addition, treatment with 1 μM lapatinib dramatically reduces (by 5-fold less; *p* < 0.04) mammosphere-forming frequency of bulk cells in the second passage. In parallel with inhibitory effect of lapatinib on BCSCs, lapatinib therapy also restrains the growth of xenograft breast tumor in mice. Twice daily oral gavage treatment by lapatinib for 14 days, results in a significant decline in tumor progression to 3.5-fold less (*p* < 0.001) in tumor size than vehicle treated tumors. Moreover, tumors from lapatinib treated mice has 50% less (*p* < 0.02) BCSCs. These mice generate 6-fold less new tumors in secondary in vivo transplantation. Lapatinib-mediated reduction of BCSC subpopulation is correlated with the inhibition of phosphorylated HER2 inside the tumors by 40% [66]. Lapatinib also reduces mammosphere-formation and proliferation of BCSCs in both HER2+ and HER2-normal ductal carcinoma in situ (DCIS) cell lines as well as in DCIS cells derived from patient samples. Lapatinib also reduces acini size of HER2+ DCIS cells in 3D matrigel culture via suppressing cell proliferation. This suggests that lapatinib does not suppress BCSC self-renewal, but may inhibit proliferation of differentiated tumor cells regardless of HER2 status [67]. A recent study reports that a lapatinib-resistant oral squamous cell carcinoma cell line SAS develops sensitivity to lapatinib during sphere-formation through the activation of HER2/AKT/Cyclin D2 pathway [68]. Induced lapatinib resistance in HER2+ BC cells also shows an up-regulated Snail and Vimentin and down-regulated E-cadherin, therefor increasing intrinsic EMT capability [69].

Another effective anti-HER2 agent that targets HER2+ BCSCs is trastuzumab. Trastuzumab (trade name Herceptin^®^, Genentech, South San Francisco, CA, USA) is a fully humanized anti-HER2 monoclonal antibody approved by Food and Drug Administration (FDA) for the treatment of HER2+ BC [70,71]. Trastuzumab binds to domain IV of HER2 and is thought to block binding pocket for receptor homo-dimerization, thereby blocking HER2 homo-dimerization, phosphorylation and consequently inhibition of downstream signaling pathways [70,71]. Following mechanisms have been suggested for the tumor inhibitory effects of trastuzumab. (i) Trastuzumab binding to HER2 suppresses PI3K/AKT and MAPK pathways by inhibition of HER2 activation [72]. In this model, trastuzumab binding to HER2 may prevent tyrosine kinase Src signaling and up-regulates activity of the tumor suppressor PTEN [73,74]. This inhibition also leads to suppression of PI3K/AKT signaling, activation of the tumor suppressor p27 and suppression of CDK2 thus arresting cell cycle and growth in BC cells [75,76,77]; (ii) Trastuzumab causes endocytosis and degradation of HER2 through blocking the activity of tyrosine kinases [78]; (iii) Preclinical and clinical studies revealed that coating HER2 overexpressed tumor cells by trastuzumab summons more immune cells especially natural killer cells to attack tumor by antibody-dependent cellular cytotoxicity (ADCC) mechanism [79,80]. Many clinical trial studies have demonstrated effectiveness of trastuzumab in combination with docetaxel in HER2+ metastatic BCs [81,82,83,84]. However, the exact mode of action and resistance mechanism still remain ambiguous. Trastuzumab treatment ameliorates disease free survival (DFS) and overall survival (OS) of patients with early stage BC and with metastatic BC and reduces the recurrence rate by almost 50% in these patients [81,82,83,84,85,86,87,88,89]. It seems that metastatic HER2+ BCSC-enriched breast tumors do not respond well to conventional chemotherapy. A retrospective analysis revealed that chemotherapy combination with trastuzumab reduced cancer relapse in 5 of 18 (27%) patients with HER2+ BCSC-enriched tumors compared to the patients who received only chemotherapy (*p* = 0.019). This result indicates that trastuzumab therapy reduces metastasis by 2.4-fold in these patients. Trastuzumab also improved OS rate of HER2+ BCSC-enriched BC patients (by 2.9-fold; *p* = 0.008), but had no significant effect on the survival of patients with HER2− or with low BCSCs tumors [58]. Further, trastuzumab decreases the percentage of CD44+/CD24− phenotype, ALDH+ cells, and mammosphere counts in luminal mammary carcinoma cell but not in basal/claudin-low cells. Injection of HER2+ BCSCs to NOD/SCID mice generated bigger tumors in a shorter period compared with HER2- BCSCs mice group. Interestingly, treating HER2+ tumors with single-agent trastuzumab immediately after tumor inoculation (early-treatment), results in significant decrease in tumor size when compared with administration after the establishment of tumors (late treatment). These data indicate that trastuzumab may inhibit the tumor growth by targeting cancer stem cells [56].

## 5. EMT Drives Resistance to Trastuzumab

EMT is a complex biologic process by which epithelial cells that are normally lied on basement membrane undergoes several gene expression reprogramming, leading to the loss of cell polarity and cell-cell adherent junctions, and the gain of mesenchymal stem cells properties with ability to migrate. EMT is necessary for three physiological and pathological processes including (i) embryogenesis, and organ developmental processes (ii) tissue regeneration and organ fibrosis; (iii) cancer migration and metastasis [90]. EMT requires cooperation of a complex cellular possesses including certain transcriptional regulatory factors including Snail, Slug, Zinc finger E-box-binding homeobox-1 (ZEB1), ZEB2, Forkhead box protein 1 (FOXC1), FOXC2, Transcription factor 3 (TCF3) and homeobox protein Goosecoid (GSC), activity of tyrosine kinase receptors, a network of several stemness signaling pathways such as TGF-β, Wnt/β-catenin, Notch, JAK/STAT, Hedgehog and also inflammatory pathways such as NF-κB, extracellular and intracellular growth factors such as Epidermal growth factors (EGFs), Insulin-like growth factor 1 (IGF1), Fibroblast growth factors (FGFs), Platelet-derived growth factor (PDGF) and interleukin-6 (IL6) and IL8, cell adhesion transmembrane proteins such as E and N-cadherins and filament protein vimentin. These processes reprogram epithelial cells to transition to the cells with more mesenchymal phenotype [91].

Liu et al. [92] demonstrate that CD44+/CD24− cells are mesenchymal-like BCSCs that localized at the tumor invasive margins and are correspond to migration and metastasis, whereas ALDH+ cells are defined as epithelial-like BCSCs that are located in deeper sites of the tumors and exhibit more proliferative property. CD44+/CD24− cells are isolated by FACS from non-tumorigenic human mammary epithelial cells that have undergone an induced EMT and exhibit many properties of BCSCs including mammosphere-formation ability [92]. On the other sides, CD44+/CD24− BCSCs isolated form breast tumors express a low level of E-cadherin, but high levels of EMT markers including N-cadherin, Vimentin, Fibronectin , ZEB1/2, FOXC2, Snail, Slug and Twist1/2 [92]. Clinical evidences reveal that HER2+ metastatic BCs are associated with EMT [93,94]. HER2 signaling in human mammary epithelial cells results in increased expression of Vimentin, N-cadherin, and Integrin-α5, as well as the loss of E-cadherin and Desmoplakin. However, some recent study suggests that loss of E-cadherin is not essential for HER2-induced EMT [95,96]. Overexpression of HER2 in epithelial BC cell line D492 induces EMT and maintains the mesenchymal phenotype in the absence of EGFR [97]. Significant evidence reveals that crosstalk between HER2 and its downstream pathways with the stemness signaling pathways prones mammary epithelial cells towards EMT.

Response to trastuzumab in luminal cells and resistance to trastuzumab in basal/mesenchymal cells may link to EMT. Trastuzumab-resistant tumors are thought to be enriched for EMT features. Basal BC cell line JIMT-1 cells are HER2+, trastuzumab-refractory, ER−, and Vimentin+. The de novo resistance of JIMT-1 cell to trastuzumab can be explained by the emergence of trastuzumab-resistance BCSCs due to the dynamic interaction between HER2 and EMT [98,99,100,101,102,103,104]. JIMT-1 cell line is composed of approximately 10% CD44+/CD24− BCSC. This level could rise to 85% at the late-passages (>60) [104]. Concurrently, HER2 expression is significantly reduced in late-passage cultures when compared to low-passage cultures. High passage JIMT-1 cells that were enriched mesenchymal CD44+/CD24− BCSCs expressing lower level of HER2 also exhibited a highly-migratogenic phenotype and produced pro-invasive/metastatic proteins more than low-passage JIMT-1 cells culture [104]. Treatment of CD44+/CD24− BCSCs derived from breast tumor tissues with formestane, an aromatase inhibitor, results in a 16% (*p* < 0.01) decrease in cell proliferation in response to single-agent trastuzumab and 50% decrease (*p* < 0.001) in response to combined trastuzumab and formestane treatment. The combined treatment also inhibits the expression of EGFR, HER2, Aromatase and Cyclin D1 in CD44+/CD24− cells, which suggests that targeting HER2 by trastuzumab may inhibit the growth of CD44+/CD24− BCSCs through the inhibition of cell cycle progression [101]. Some other reports suggest that preferential killing of the putative CD44+/CD24− BCSCs might be sufficient to overcome primary resistance to trastuzumab. The CD44+/CD24− BCSCs derived from trastuzumab-refractory JIMT-1 cells were 10-fold more sensitive to cell growth inhibitory effects of metformin than the other cells [103]. JIMT-1 cell line is highly enriched with the mesenchymal phenotype CD44+/CD24− fraction in late passages [104,105,106]. Indeed, treatment of JIMT-1 tumors with trastuzumab fails to exhibit significant reductions in tumor volume but when trastuzumab is combined with metformin, the tumor size is significantly smaller than those of the groups treated with single-agent trastuzumab or metformin [103]. These results suggest that BCSCs escape from trastuzumab effects, which may be a mechanism of resistance to trastuzumab.

It is further revealed that trastuzumab-resistant HER2+ cells show spontaneous EMT and predominant exhibition of CD44+/CD24− [107]. There is a synchronous increase in CD44 and elements of Wnt/β-catenin signaling and a decrease in CD24 expression in mesenchymal colony clusters of SKBR-3. SKBR-3 cell is characterized as HER2+/trastuzumab-sensitive cell line. The CD44+/CD24− mesenchymal colonies of SKBR-3 also show significant up-regulated EMT markers including Vimentin, N-cadherin, Twist1 and Fibronectin. The colonies are resistant to trastuzumab and lapatinib while the luminal SKBR-3 cells remain trastuzumab-sensitive. Similar to previous reports, HER2 expression levels in mesenchymal colonies are negatively correlated with trastuzumab resistance [107]. Thus, lapatinib may not be a useful therapeutic option in targeting CD44+/CD24− mesenchymal cell rich tumors due to the negative regulation of HER2 during EMT process.

The expression of the EMT-driving transcription factors Slug, Twist1 and ZEB1 is higher in trastuzumab-refractory basal HER2+ JIMT-1 cells than that in the trastuzumab-responsive luminal HER2+ SKBR-3 cells. The knockdown of these three transcription factors in parental JIMT-1 cells reduces the subpopulation of CD44+/CD24− BCSC by 5, 5 and 2-fold, respectively. Interestingly, depletion of the EMT-driving transcription factors increases the trastuzumab-refractory in JIMT-1 tumors due to sensitized CD44+/CD24− BCSCs inside the bulk JIMT-1 tumors [102]. HER2+ BCSCs are susceptible to change in their expression signature during EMT. This phenomenon may explain resistance of some HER2+ breast tumors to HER2-targeting agent including trastuzumab. Further, CD44+/CD24− phenotype HER2+ BCSCs may escape from trastuzumab-mediated ADCC. BCSCs could survive immunoselection process in BC cells co-cultured with NK cells and trastuzumab. This resistance may be attributed to the reduced HER2 expression levels on their surface [108].

The authentic mechanism of de novo resistance to trastuzumab is linked to down-regulation of HER2 extracellular domain due to HER2 shedding. HER2 shedding is characterized by the cleavage of HER2 extracellular domain that is responsible for binding trastuzumab, resulting in a receptor with only transmembrane and intracellular domain. The cleaved HER2 receptor still possesses tyrosine kinase activity [109,110,111]. HER2 shedding takes place by proteolytic cleavage of full length HER2 (p185HER2) from juxtamembrane region of HER2 by zinc-containing metalloproteinase, including A disintegrin and metalloproteinases (ADAM) and matrix metalloproteinases (MMPs) family members [112,113,114,115]. Interestingly, up-regulation of the metalloproteinases is a hallmark of EMT and mesenchymal cells [116,117,118,119]. Several metalloproteinases including ADAMs 9, 10, 12, 15, 17, 28 [120,121,122,123,124,125] and also MMPs 1, 2, 7, 9 11, 12, 13, 14 and 16 are reported as overactivated in many BCs [126,127,128,129]. ADAMs 10 and 17 are the major sheddase enzymes involved in HER2 shedding [130]. Recent studies show that the metalloproteinases are also associated with poorer relapse-free survival in HER2+ BC patients and the inhibition of metalloproteinases by chemicals overcame trastuzumab resistance in both naïve and trastuzumab-resistant HER2+ cell lines [131,132,133]. These data suggest ADAMs 10 and 17 as key drivers of trastuzumab resistance and potential targets to overcome trastuzumab resistance in HER2+ BC [134]. Moreover, a preclinical study revealed that inhibitors of MMP1, MMP2, MMP3 and MMP9 suppress HER2 shedding [135]. HER2 shedding also results in the production of kinase-active p95HER2. Many cohort studies conclusively show the correlation of p95HER2 expression with poor prognosis and trastuzumab resistance in BC, corroborating p95HER2 as prognostic factor for metastasis and a predictive marker of trastuzumab resistance [110,136,137,138,139]. Taking together, increased metalloproteinases activity during EMT may allow HER2 to escape the inhibition by trastuzumab, and thus lead to resistance to trastuzumab in BC.

## 6. HER2 Promotes Stemness Signaling Pathways

### 6.1. TGF-β/Smad Signaling

Transforming growth factor-β (TGF-β) superfamily signaling plays critical roles in embryo development, adult tissue regeneration and tumorigenesis by regulating cell growth, differentiation, apoptosis, cellular homeostasis and other cellular functions [140]. TGF-β is encoded by 33 genes that produce different structurally related polypeptides correspond to pleiotropic cytokine ligands [141,142]. Signaling by TGF-β is transduced through binding TGF-β ligands to type II cell surface receptors that are serine/threonine receptor kinase and catalyzes the phosphorylation of type I receptors [143,144]. A mammalian cell utilizes seven known type I receptors that called activing receptor like kinases (ALK)-1 to 7 and five type II receptors called as TGF-β type II receptor (TβRII), Activin type II receptor (ActRII), Activin type II receptor B (ActRIIB), BMP type II receptor (BMPRII), and Anti-Müllerian hormone receptor (AMHR). Among the ligands, TGF-β activates ALK5, Activin activates ALK4, Nodal activates ALK4 and ALK7 and BMPs activate ALK1, ALK2, ALK3 and ALK6 [145]. Activation of type I receptors induces phosphorylation of downstream signal transducer receptor-activated Smads (R-Smads). Phosphorylated R-Smads form a heteroligomeric complexes with Smad4 (Co-Smad). The Smad complexes translocate into the nucleus and regulates the expression of target genes by direct binding to the target DNA and/or via interaction with various transcriptional cofactors depending on the status of the cell [145]. TGF-β can also drive several non-Smad signaling pathways including ERK, p38 kinase, c-JUN N-terminal kinase (JNK), PI3K/AKT, RhoA, Rac1, and Cdc42 GTPases [146].

The role of the TGF-β signaling pathway in growth, apoptosis, self-renewal and differentiation of stem cells/progenitor cells has been convincingly demonstrated [140]. It is well known that Smad and non-Smad pathways play critical role in stemness and act as an inducer of EMT of normal mammary epithelial cells [147,148,149]. TGF-β signaling promotes tumor cell proliferation, survival, motility, invasion, metastatic colonization and acquisition of mesenchymal markers, such as increased Fibronectin and Vimentin expression, increased invasiveness, and exhibiting CD44+/CD24− phenotype toward BCSC progression straits through induction of EMT [150,151,152]. TGF-β/Smad signaling increases the expression of transcription factors and transcription regulators involved in EMT, including Snail [153], Slug [154], ZEB1 [152] ZEB2 [155], High mobility group A2 (HMGA2) [156] and ETS1 [157]. TGF-β/Smads signaling also suppresses E-cadherin by up-regulating mesenchymal phenotype and by down-regulating the Inhibitor of differentiation (Id)-1, 2, and 3 proteins that are the negative regulators of the TGF-β-induced ZEB1 and ZEB2 [158,159]. Smad2 can suppress epithelial markers E-cadherin, Claudin-4, Kallikrein-10, and Cingulin by activation of DNA methyltransferase 1 (DNMT1)-mediated epigenetic silencing of the corresponding genes [160]. There are more evidence supporting epigenetic modification of EMT by TGF-β. The microRNA-200 (miR-200) family members have been shown to increase E-cadherin expression and to alter the cancer cell morphology to an epithelial phenotype by predominantly down-regulating TGF-β and ZEBs. In a reverse loop, TGF-β-mediated ZEB1 activation inhibits transcription of miR-200 family members resulting in suppressed E-cadherin and increased Vimentin [161,162,163,164]. Additionally, Smad3 can form complex with Myocardin-related transcription factors (MRTFs), which leads to nuclear translocation of MRTFs promoting expression of Slug [165].

In addition to Smads-dependent pathways of TGF-β signaling, non-Smad pathways induced by TGF-β such as PI3K/AKT, MAPK [146,166], RhoA, and Cofilin are also involved in promoting EMT [167]. TGF-β activates mammalian TOR complex 1 (mTORC1) and mTORC2 through PI3K/AKT pathway [168,169,170]. AKT increases the expression level of Snail and MMP9. AKT also up-regulates Snail via the phosphorylation and inactivation of glycogen synthase kinase 3 (GSK3), a serine-threonine kinase [171,172]. TGF-β-induced AKT phosphorylation releases heterogeneous nuclear ribonucleoprotein E1 (hnRNPE1) from the 3′ untranslated regions of Disabled 2 (DAB2) and Interleukin (IL)-like EMT inducer mRNA and allows progression of EMT [173]. TGF-β ligands also promote the p38, JNK and MAPK signaling pathways [174]. TGF-β-induced MAPK signaling inhibits GSK3, therefore stabilizes the activity of Snail [175]. Besides, active oncogenic Ras signaling positively regulates the induction of Snail by TGF-β [176]. Cooperation between the TGF-β and MAPK pathways also causes emerging CD24− stem cell-like cells from CD24+ differentiated cells, which suggests b a role for TGF-β in EMT and the exhibition of CD24− phenotype [177]. In addition, TGF-β signaling retains mesenchymal state of CD44+/CD24−/ALDH+ cells and their tumorigenicity after TGF-β-induced EMT [178,179]. Moreover, TGF-β induces JNK phosphorylation, transactivation of c-JUN and Activator protein-1 (AP1) complex, which leads to EMT [180]. Other studies show that TGF-β-mediated EMT requires the activation of RhoA, a positive regulator of actin cytoskeleton and cadherin junctions in cell-cell contact [181,182].

**Figure 1:**
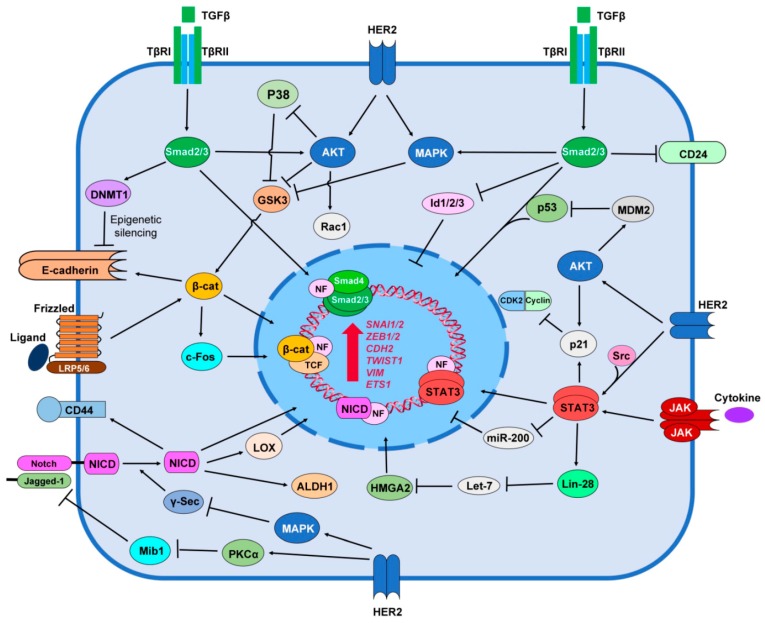
Crosstalk between HER2 and BC stemness signaling pathways. Active TGF-β/Smad induces EMT by direct regulation of transcription of EMT genes and indirectly by activating DNMT1, inactivating Id1/2/3 and GSK3 through AKT and MAPK. HER2 regulates TGF-β/Smad-mediated induction of EMT through activating AKT, MAPK, and the p53 inhibitor MDM2. Active Wnt/β-catenin signaling regulates EMT by β-catenin-mediated transcriptional regulation of EMT genes through activating c-Fos. HER2-mediated activation of AKT and MAPK inhibits β-catenin through inhibition of GSK3. Notch signaling induces EMT directly via transcriptional regulation of EMT genes by NICD. NICD also up-regulates LOX and ALDH. HER2-mediated activation of MAPK inhibits cleavage of Notch receptor by inhibition of γ-secretase. In addition, HER2 itself positively affects the activation of Notch by activating PKCα that is an inhibitor of Mib1. Inhibition of Mib1 causes up-regulation of Jagged-1 a ligand for Notch receptor. Active STAT proteins up-regulate expression of EMT genes directly. STATs also up-regulate EMT by three indirect paths including inhibiting mir-200 via activating Lin-28 that inhibits Let-7. Let-7 in turn inhibits HMGA2, an EMT marker. STATs also activate p21 that down-regulates CDK2. HER2 interacts with these axes by phosphorylating and activating STATs through Src and by activating p21 through AKT. Direct and indirect interactions are not distinguished in the figure. NF: nuclear factor.

Accumulating evidence also indicates a functional crosstalk between HER2 tyrosine kinase and the TGF-β signaling. In HER2-overexpressing BC, this crosstalk results in increased cancer cell proliferation, survival and invasion, accelerated cancer progression and metastasis in animal models, as well as resistance to chemotherapy and HER2-targeted therapy. Studies indicate that interaction between HER2 and TGF-β takes place in several levels including; (i) suppression of Smad-dependent transcriptional regulation and its downstream target genes by HER2; (ii) activation of the HER2 downstream pathways (PI3K/AKT and MAPK pathways) by TGF-β in a Smad-independent manner and (iii) modification of the tumor microenvironment by secretory mediators that are regulated by both downstream mediators of HER2 and TGF-β receptors [183] (Figure 1). There is a cooperation between HER2 and TGF-β in BC development. Ueda et al. [184] shows that exogenous TGF-β ligand and ectopic expression of TGF-β type I receptor ALK5 activates TGF-β signaling and induced motility in HER2-overexpessing MCF10A cells (MCF10A/HER2). Moreover, inhibition of HER2, PI3K/AKT, MAPK, and Integrin β1 all abrogate TGF-β-induced motility in MCF10A/HER2 cells. In addition, trastuzumab blocks TGF-β-stimulated Rac1 activation in the HER2-overexpessing cells, which suggests that HER2 and TGF-β crosstalk with each other to regulate tumor cell motility [184]. Overexpression of either TGF-β1 or ALK5 in HER2+ BC xenograft tumors reduces apoptosis, but increases survival, angiogenesis, local invasion, metastasis [185,186,187]. The ability of HER2 to cooperates with TGF-β is correlated with higher levels of active Smad2, AKT, MAPK and p38, as well as Vimentin [186,187]. As TGF-β can activate PI3K/AKT and MAPK pathways independent of Smad, it seems that HER2 and TGF-β utilize common paths to promote tumor cell invasion.

Moreover, the interaction between HER2 and TGF-β regulates DNA repair and the resistance to DNA-damaging chemotherapy in cancer cells. TGF-β/Smad signaling requires p53 to regulate MutS homolog 2 (MSH2), a key component of the DNA mismatch repair (MMR) system. Obviously, this function of TGF-β is impaired in the absence of p53, a frequent mutation in BCs. On the other hands, through PI3K/AKT pathway, HER2 down-regulates p53 signaling by inducing nuclear translocation of MDM2, an E3 ubiquitin ligase that targets p53 [188]. HER2 also abrogate p53-mediated transcriptional regulation of MSH2 in p53-proficient BC cells by increasing the expression level of miR-21 via TGF-β [189]. Blockade of HER2-TGF-β crosstalk may significantly enhance the efficiency of conventional therapies in BC patients with HER2 overexpression [190]. In summary, in HER2-overexpressing BC, crosstalk between HER2 and TGF-β results in increased cancer cell proliferation, survival and invasion, accelerated metastasis in animal models, resistance to chemotherapy and HER2-targeted therapy and perhaps up-regulation of BCSCs.

### 6.2. Notch Signaling

Notch signaling is an evolutionarily conserved pathway that acts as a mediator of short-range cell-cell communication and is present in most multicellular organisms. Notch signaling regulates multiple aspects of invertebrate and vertebrate cell fate determination during development and maintains adult tissue homeostasis. Like TGF-β signaling, Notch signaling is an essential process for self-renewal, differentiation and is critical in multiple stages of development, in lineage-specific differentiation of pluripotent embryonic stem cells, and in controlling stem cell population and activity in the context of tissue degeneration, regeneration, and malignancy [191]. Notch proteins are cell surface transmembrane-spanning receptors that are normally activated by ligand binding during direct cell-to-cell contact [192,193]. The extracellular domain of all Notch proteins contains 29–36 tandem EGF-like repeats that interact with the Delta, Serrate, and Lag2 (called DSL) domain of ligands from neighboring cell [193,194,195]. In mammals, there are four Notch receptors (Notch1-4) and five canonical ligands [191]. Interaction between Notch receptor and Notch ligands initiates proteolytic cleavage of the receptor by metalloproteinases. The cleavage of Notch receptor by γ-secretase causes the release of the Notch intracellular domain (NICD) [196,197,198]. Upon intracellular cleavage, the NICD translocates to the nucleus and interacts with the CSL (CBF1, Suppressor of Hairless, Lag1) family of DNA-binding proteins to form a transcriptional activator complex, which regulate the expression of target genes [196,197,198].

Dontu and colleagues [199] have demonstrated the critical role of Notch signaling in maintaining normal human mammary stemness by increasing self-renewal efficiency. Up-regulated Notch signaling increases the self-renewal and transformation of luminal mammary stem cells, leading to hyperplasia and tumorigenesis [200]. It is well-demonstrated that the Notch signaling has a regulatory role in breast tumorigenesis, metastasis and resistance. Notch signaling maintains BC stemness by promoting BCSC phenotype and EMT. Inhibition of Notch signaling by Notch4 neutralizing antibody or γ-secretase inhibitors (GSIs) suppresses BCSC subpopulation and blocks mammosphere-formation effectively [199,201,202,203]. Notch3 is also a positive factor in self-renewal of BCSC mammospheres [204,205]. Activated Notch signaling increase ALDH1 activity and promotes BC stemness through induction of deacetylase Sirtuin 2 (SIRT2), an enzyme that deacetylates and activates ALDH1 [206]. Whereas, inhibition of Notch activity in the cells by glucose functionalized nanoparticles containing GSIs reduces pool of ALDH1+ BCSCs [207]. Notch signaling in epithelial BC cell line MCF-7 reduces the expression of estrogen receptors and increases CD44 expression in vitro and in vivo models. Moreover, inhibition of Notch1 with a GSI, DAPT, and shRNA reduces the expression of CD44+/CD24− phenotype, matrigel invasion and micro- and macrometastases [208,209]. Radioresistance of CD44+/CD24− cells derived from MCF-7 and MDA-MB-231 BC monolayer cultures is correlated with high expression of Notch1 in the BCSCs, implying association of Notch pathway with stemness-related resistance [209]. Recently, it is found that Notch signaling is a critical regulator of breast tumor EMT by ionizing radiation. During radiation, induced Notch2 accelerates tumor malignancy by increasing mesenchymal markers through IL6/JAK/STAT3 signaling axis [210]. However, CD44+/CD24+ but not CD44+/CD24− cells in TNBC cell lines express activated Notch1 intracellular domain (NICD1) and its target genes [211]. GSI reduces mammosphere-formation and tumor growth of CD44+/CD24+ cells, but not CD44+/CD24− cells [212].

As pointed out above, Notch signaling pathway has been known as an important regulator of EMT induction. Timmerman et al. [213] demonstrates that Notch signaling activity promotes EMT during both cardiac development and oncogenic transformation by transcriptional induction of the Snail and repression of E-cadherin expression. Notch signaling-mediated EMT takes place by down-regulation of endothelial markers and up-regulation of mesenchymal markers [212]. Slug has also has been reported as a direct target for Notch pathway. Notch signaling can up-regulate expression of Slug and Snail either directly or indirectly through interaction with TGF-β signaling [214,215,216]. Moreover, during hypoxia-induced EMT, NICD1 activates the expression of Snail directly by regulating Snail mRNA and indirectly via up-regulation of Lysyl oxidase (LOX) that stabilizes the Snail protein [217].

Notch signaling may support breast tumorigenesis by promoting cell growth and survival and by inhibiting differentiation. Cooporation among Notch1, PI3K/AKT, and MAPK pathways has been demonstrated in development of BC [218,219]. It seems that Notch up-regulates HER2 but HER2 down-regulates Notch signaling (Figure 1). HER2+ BC cells have low Notch signaling activity and inhibition of HER2 by trastuzumab increases nuclear localization of Notch1 and the expression of genes targetted by Notch pathway [220]. The mechanism by which HER2 down-regulates Notch signaling is not clear. A study shows that HER2/MAPK pathway suppresses activity of the γ-secretase complex, and thus results in reduced levels of Notch1 cleavage and NICD1 expression [221]. Pandaya et al. [222] recently showed that HER2 may limit ubiquitinylation of Jagged1, by suppressing expression of Mindbomb1 (Mib1), an E3 ligase, and by activating Protein kinase C-α (PKCα) that negatively regulates the interaction between Mib1 and Jagged1. Finally, since trastuzumab resistant cells show high level of Notch activity, inhibition of Notch pathway by GSIs overcame trstuzumab resistance in these cells [220]. Moreover, trastuzumab threatment induces the activation of Notch signaling. Thus a combined inhibition of HER2 and Notch signalling (trastuzumab plus GSIs) has better outcome in both trastuzumab-resistant and sensitive HER2+ BC tumors [220,222,223].

### 6.3. Wnt/β-catenin Signaling

The Wnt/β-catenin pathway is a conserved pathway that regulates crucial aspects of cell fate decisions, cell migration, cell polarity, stem cell pluripotency, and neural patterning. Wnt/β-catenin signaling is initiated through binding Wnt ligands to two distinct receptor families including Frizzled (Fz) family of transmembrane receptor proteins and lipoprotein receptor-related proteins 5 and 6 (LRP5/6) [224,225]. In human, there are seven Fz receptors and nineteen cysteine rich Wnt glycoprotein ligands with highly conserved approximately 350–400 amino acids [226]. Wnt receptor activation initiates canonical (β-catenin-dependent) and non-canonical (β-catenin-independent) pathways. In canonical signaling pathway formation of ligand-receptor complex activates kinase domain of the receptor that causes phosphorylation of serine residues in the cytoplasmic tail of LRP5/6. Phosphorylated LRP5/6 recruits scaffolding protein Axin. Axin is a necessary component of a multi-protein complex that degrades β-catenin [227]. This β-catenin destruction complex also includes Adenomatosis polyposis coli (APC), Protein phosphatase 2A (PP2A), GSK3 and Casein kinase 1α (CK1α) [228,229,230]. In the absence of Wnt ligand, Axin contributes to the formation of β-catenin destruction complex, which leads to phosphorylation of β-catenin on serine and threonine residues near its N-terminus providing β-catenin a target for ubiquitination and rendering it to ubiquitin-dependent proteasome-mediated degradation [228,229,230]. With active signal, restraining Axin by LRP5/6 prohibits formation of β-catenin destruction complex and causes an accumulation of β-catenin in the cytoplasm and its eventual translocation into the nucleus [227,231]. In the nucleus, β-catenin acts as a transcriptional coactivator and forms a complex with members of the T-cell factor/lymphoid enhancing factor (LEF/TCF) family of DNA binding proteins that regulates transcription of target genes [232,233]. β-catenin is also involved in the regulation and coordination of cell–cell adhesion by enhancing the association of adherens junction complex with E-cadherin, an essential process for the maintenance of epithelial cell layer [227].

The driving role of Wnt/β-catenin signaling pathway has been well-defined in the development of many human cancers including BC, and appear to be associated with cancer stem cell biology. Several studies in mice have revealed that Wnt/β-catenin signaling controls mammary gland development and differentiation during embryogenesis and is critical for stem cell maintenance inside mammary tissue [234,235]. Wnt/β-catenin signaling determines the developmental fate of mammary gland stem cells by regulating mammary epithelium [236]. Studies in both mouse models and human BCs have shown that active Wnt/β-catenin signaling is essential for breast tumorigenesis. Wnt/β-catenin signaling is higher in BCSCs than in normal stem cells [237]. Inhibition of β-catenin suppresses stemness activity in patient-derived metastatic BC, implicating important role of the Wnt/β-catenin signaling in BCSCs [237,238]. Whereas, activated Wnt/β-catenin signaling are associated with increased stemness activity and radiation resistance of BCSCs [239].

Expression of Wnt3 in ER− BCs increases the mammosphere-forming ability [237]. Expression of Wnt3 in trastuzumab-resistant cells also increases the expression of EMT markers including N-cadherin, Twist1, Slug, and decreases epithelial marker E-cadherin [240]. In normal mammary epithelial cells c-Fos oncogene decreases E-cadherin and induces EMT through Wnt/β-catenin signaling [241]. β-catenin itself has been shown to induce EMT via induction of LEF1 expression [242]. Wnt receptor LGR5 has been shown to be a stemness marker for mammary gland and essential for postnatal mammary gland organogenesis [243]. BCSCs with high level LGR5 expression form more mammospheres and are more potent to drive BC progression and metastasis [244]. LGR5 expression in BC increases cell mobility, tumor growth, pulmonary metastasis, and mammosphere-formation and stemness properties of BC cells through Wnt/β-catenin-induced EMT [245]. LGR5 potentiates Wnt/β-catenin pathway in BCSCs and is required for the maintenance of spheroid-derived CD44+/CD24− BCSCs [245]. During EMT, β-catenin binds to Twist1 to increases the transcriptional activity of β-catenin/TCF4 complex by binding to the promoter DNA of ABCG2, a cancer stemness marker [246].

Some reports suggest a friend and foe relationship between HER2 and Wnt/β-catenin signaling pathway in breast tumor cells. Schroeder et al. [247] reports that HER2 makes a complexe with both membranous and cytoplasmic β-catenin protein to induce phosphorylation of β-catenin in ductal BC tissues but not in normal mammary tissues. Wang et al. [248] shows that destabilization of HER2 receptor by Heat shock protein-90 kDa (HSP-90) inhibitor geldanamycin disrupts association of HER2 with β-catenin and suppresses Wnt/β-catenin signaling pathway. Geldanamycin-mediated inhibition of HER2 also attenuates HER2+ BC cell proliferation and motility via suppression of Wnt/β-catenin [248]. HER2 influences Wnt/β-catenin signaling through its downstream regulators AKT and MAPK (Figure 1). These regulators can inhibit GSK-3 that leads to translocation of β-catenin to the nucleus to promote transcription of β-catenin-TCF target genes [249]. Expression of nucleocytoplasmic β-catenin is significantly abundant in HER2 expressing node-positive breast carcinomas when compared with HER2− node-positive tumors. Nucleocytoplasmic β-catenin expression was also higher in transgene HER2+ murine mammary DCIS tumors [238]. Wnt3 ligand-mediated activation of Wnt/β-catenin pathway induces EMT and reduces sensitivity to trastuzumab in HER2+ BC cells [240]. According to this report, approximately 95% (22 genes) of Wnt/β-catenin signaling genes are regulated in trastuzumab resistant HER2+ BC. Of 22 genes, 11 genes are up-regulated and 11 genes are down-regulated, which suggests that Wnt/β-catenin/TCF axis may drive trastuzumab resistance via regulating EMT [240]. As mentioned above one of the characteristics of EMT is the up-regulation of MPs. Thus, it is likely that the resistance to trastuzumab is due to HER2 cleavage and lose of HER2 extracellular part by EMT-related MPs. These results suggest a potential negative feedback loop between HER2 and β-catenin through EMT. However, some other studies indicate that up-regulated expression of β-catenin is more common in TNBC compared to HER2+ breast tumors [250,251].

### 6.4. JAK/STAT Signaling

In human mammary tissue JAK/STAT signaling pathway transmits signals to the nucleus, which leads to the transcription of a wide range of genes involved in cell proliferation, differentiation, migration and apoptosis. JAK/STAK signaling controls immunity, spermatogenesis, hematopoiesis and development of mammary gland and breast tumor. JAK/STAT signaling is initiated by binding of various ligands (mostly cytokines) to a Janus kinase (JAK) receptor, which induces JAK dimerization and phosphorylation of its tyrosine residues [252,253]. This event provides a binding site for SH2 domain of Signal transducer and activator of transcription (STAT). The binding of STATs to the JAK receptors results in the phosphorylation of STATs by JAKs. Phosphorylated STATs dimerize with each other and migrate to the nucleus where the dimer regulates the transcription of target genes [254,255,256].

Activation of JAK/STAT signaling pathway is necessary for growth, proliferation, survival and chemo-resistance of CD44+/CD24− BCSCs [19,257,258]. Targeting JAK2 and/or STAT3 results in a reduction of the CD44+/CD24− subpopulation and in vivo tumorigenicity of BC cells, which suggests that JAK/STAT signaling plays important role in BCSC maintenance in basal-like tumors [19]. In patient-derived Claudin-low BC cells STAT3 activity is associated with increased mammosphere-forming efficiency and tumorigenicity [258]. High STAT proteins level is also found in CD44+/CD24− and ALDH+ BCSCs. Inhibition of STAT3 by shRNA reduces the viability and mammosphere-forming ability of BC cells [259]. In addition, selective inhibition of STAT3 by small molecule inhibitors suppresses CD44+/CD24−/ALDH+ BCSCs, mammosphere-forming efficiency and tumor growth in human breast tumor xenograft rodents [260]. Moreover, targeting CD44 in basal-like BC cells leads to repression of JAK/STAT signaling as well as invasive markers MPs [261]. In addition, epigenomics analysis of BCSCs derived from mammospheres reveals that JAK/STAT signaling is associated with the exhibition of CD44+/CD24− cancer stemness phonotype [262].

JAK/STAT signaling has been shown to play important role in BC EMT induction. IL6 induces the expression of Twist1 via activating STAT3 in MCF-7 cell line [263]. Additionally, exposure of JAK to IL6 increases the population of CD44+ BCSC through inducing STAT3-mediated EMT of epithelial-like BC cells [264]. Oncostatin M (OSM), another inducer of JAK/STAT signaling, is expressed in an autocrine/paracrine fashion during EMT of breast tumor cell. OSM has been shown to enhance cell migration and up-regulate the expression of EMT inducers including extracellular matrix (ECM) protein and Fibronectin in mammary epithelial cells through STAT3 [265,266]. OSM-mediated activation of STAT3 is also able to up-regulate EMT by down-regulating miR-200. During BC EMT, STAT3 also promotes the transcription of Lin28, resulting in the down-regulation of let7and the up-regulation of mediator High-mobility group A protein 2 (HMGA2) [267]. EGF-mediated induction of JAK/STAT3 signaling is also able to induce BC EMT via up-regulating Twist1 [266]. Moreover, Transient receptor potential-melastatin-like 7 (TRPM7) channel up-regulates the expression of Vimentin through increasing EGF-induced STAT3 activation, which suggests importance of EGF-STAT3-TRPM7 in regulation of calcium-dependent EMT in BC [268].

HER2 regulates STAT-mediated induction of BC EMT and stemness (Figure 1). The heterodimerization between HER2 and HER4 leads to the activation of Src kinase, which stimulates JAK/STAT5 signaling pathway [269]. HER2 dimerization induces phosphorylation, dimerization and nuclear translocation of STAT3 in a Src-dependent manner [270]. HER2 exerts some other functions through JAK/STAT3 signaling. HER2-mediated activity of Src further activates STAT3 that up-regulates transcriptional expression of p21^Cip1^, a cyclin-dependent kinase inhibitor [271]. Silencing STAT3 in HER2+ BC cells reduces tumor invasion suggesting a cooperation between HER2 and STAT3 in tumorigenesis [272]. In HER2+ BC, HER2 increases STAT3 activation and expression of STAT3 target genes including MPs in an autocrine manner by inducing IL6 secretion [273]. It is possible that HER2/IL6/STAT3 signaling axis drives EMT by up-regulating MPs. Phosphorylated STAT3 in HER2-overexpressing BC cell lines promotes the stem-like cell and EMT phenotype by up-regulating Oct4, Sox2, CD44 and Slug. While activation of STAT3 in HER2 overexpression cells also increases the mammosphere-formation efficiency, inhibition of HER2 and/or STAT3 abolishes BCSC and EMT phenotype. These data suggest a possible cooperation between HER2 and JAK/STAT signaling in emergence of BCSCs [274].

### 6.5. Hedgehog Signaling

The Hedgehog (Hh) signaling pathway regulates embryogenesis, organogenesis and adult tissue maintenance by controlling cell proliferation, renewal, differentiation, cell motility and adhesion as well as EMT. Aberrant activity of Hh signaling is directly linked to many human diseases including cancers. It has been reported that Hh signaling plays a key role in development of BC through transformation of adult stem cells into cancer stem cells [275]. In mammals, the canonical Hh signaling pathway is initiated by binding of three Hh ligands [276,277] to the twelve-pass transmembrane protein receptors Patched1 (Ptch1) and Patched2 (Ptch2) [276,277,278]. The three Hh ligands include Sonic Hedgehog (Shh, the most broadly expressed and best studied Hh molecule) [276], Indian Hedgehog (Ihh, primarily involved in bone differentiation) [276], and Desert Hedgehog (Dhh, involved in gonad differentiation) [278]. In the absence of Hh, Ptch1 constitutively represses GPCR-like protein Smoothened (Smo), a seven-transmembrane domain receptor [279]. Hh binding to Patch1 relieves the inhibition on Smo, which results in Smo accumulation in cilia and the phosphorylation of its cytoplasmic tail [280]. This signal facilitates the release of Glioma-associated oncogene (Gli) family of latent zinc-finger transcriptional mediators from kinesin-family proteins Kif7 and Sufu, leading to the activation and nuclear translocation of the Gli transcription factors. Gli transcription factors then regulate the transcription of the target genes [281,282,283,284].

Growing evidence suggests important role of Hh signaling pathway in maintaining BC stemness [285]. Recent clinical studies indicate that high expression of Ptch1 and Gli1 is associated with larger tumors, metastasis, pathological progression and with significantly shorter OS and DFS in BC patients with CD44+/CD24− BCSC-enriched tumors [286]. High RNA expression levels of Ptch1, Gli1 and Gli2 have been reported in CD44+/CD24−/Lin− BCSCs [287,288]. Activation of Hh signaling increases CD44+/CD24− cell population and mammosphere size. However, inhibition of Hh signaling pathway suppresses CD44+/CD24− BCSC subpopulation, mammosphere-forming and abrogates drug resistance of BCSCs [288,289,290,291]. Inhibition of Hh signaling also suppresses EMT by inhibiting Snail, Slug and ZEB2 [292]. It is recently found that salinomycin that shows selective toxicity in BCSCs, inhibits Shh-mediated Hh activation through down-regulating the expression of Ptch1, Smo, Gli1, and Gli2 as well as stemness markers Snail, Nanog, Oct4 and Sox2 [291,293,294]. Therefore, Hh signaling induces self-renewal and EMT of BC cells [292,295,296].

It is recently reported that high level expression of Shh and Gli1 is correlated with HER2 expression. Inhibition of Hedgehog acyltransferase, a key enzyme for Shh synthesis, reduces HER2+ BC growth [297,298,299]. The data regarding the crosstalk between HER2 and Hh signaling in BC are very limited. However, it is reported that HER2 downstream pathways PI3K/AKT and MAPK interact with Hh signaling pathway in regulating tumorigenesis and stemness in chronic lymphocytic leukemia [300], ovarian [301], pancreatic [302] and esophageal [303] cancers.

## 7. Concluding Remarks

**Figure 2:**
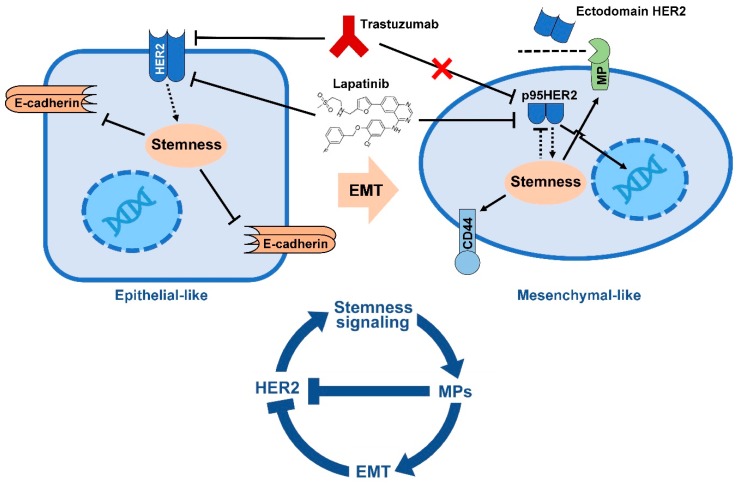
Negative feedback relationship between HER2 and BC stemness leading to trastuzumab resistance. HER2+ BC cell responds to trastuzumab and lapatinib in epithelial-like context. In this context, HER2 increases expression of stemness markers resulting in down-regulation of epithelial marker E-cadherin and up-regulation of EMT phenotype including MPs. In mesenchymal context MPs cleave HER2 receptor from juxtamembrane of the receptor that results HER2 shedding and expression of p95HER2. p95HER2 maintain its kinase activity and is able to activate AKT and MAPK pathways and to translocate to the nucleus where acts as transcription co-factor. Mesenchymal-like p95HER2+ cell respond to lapatinib but is resistant to trastuzumab.

HER2 is an important target for treatment of HER2+ BCs. Several HER2-targeting agents including trastuzumab have been approved by FDA to treat HER2 positive BC. However, the resistance to these HER2 targeting agents have become a huge obstacle for the treatment of HER2-positive BC patients. It is not clear how many HER2+ tumors developed resistance to trastuzumab. As discussed in this review, one likely mechanism could be attributed to cleavage/shedding of HER2 extracellular domain by MPs. In this review, we also described how HER2 interacts with stemness signaling such as TGF-β/Smad, Notch, Wnt/β-catenin and JAK/STAT pathways in epithelial BC cell to induce EMT and how this phenomenon leads to trastuzumab resistance (Figure 2). In summary, HER2 promotes EMT in HER2+ BC. During EMT, up-regulation of metalloproteinases is required to cleave cell-cell adhesion molecules, matrix proteins, differentiation factors and a wide range of cell surface receptors including HER2. EMT-mediated HER2 cleavage is characterized by decreased cell surface full length HER2 with extracellular domain, but increased intracellular p95HER2 that maintain kinase activity and is able to migrate to the nucleus to act as oncogenic nuclear factor. As trastuzumab exerts its anti-tumor activity by interacting with HER2 extracellular domain, it does not inhibit p95HER2. In conclusion, it is likely that a negative feedback loop between HER2, stemness signaling and EMT can explain HER2+ BC resistance to trastuzumab.

Thus, preventing this feedback loop by targeting EMT or metalloproteinases may be an important practical approach to overcome trastuzumab resistance in HER2+ tumors. In fact, various drugs targeting metalloproteinases and other EMT related molecules have been developed and test in laboratory and clinical setting [304,305]. Hence, dual inhibition of HER2 and metalloproteinases could be an effective treatment strategy to targeting HER2+ BC and overcome trastuzumab resistance. Future research should be conducted to test a combination of trastuzumab and metalloproteinase inhibitors on HER2+ BCs.

## References

[1] Weigelt B., Horlings H.M., Kreike B., Hayes M.M., Hauptmann M., Wessels L.F.A., de Jong D., Van de Vijver M.J., Van’t Veer L.J., Peterse J.L. (2008). Refinement of breast cancer classification by molecular characterization of histological special types. J. Pathol..

[2] Slamon D.J., Godolphin W., Jones L.A., Holt J.A., Wong S.G., Keith D.E., Levin W.J., Stuart S.G., Udove J., Ullrich A. (1989). Studies of the HER-2/neu proto-oncogene in human breast and ovarian cancer. Science.

[3] Slamon D.J., Clark G.M., Wong S.G., Levin W.J., Ullrich A., McGuire W.L. (1987). Human breast cancer: Correlation of relapse and survival with amplification of the HER-2/neu oncogene. Science.

[4] Tandon A.K., Clark G.M., Chamness G.C., Ullrich A., McGuire W.L. (1989). HER-2/neu oncogene protein and prognosis in breast cancer. J. Clin. Oncol..

[5] Pegram M.D., Konecny G., Slamon D.J. (2000). The molecular and cellular biology of HER2/neu gene amplification/overexpression and the clinical development of herceptin (trastuzumab) therapy for breast cancer. Cancer Treat. Res..

[6] Soady K.J., Kendrick H., Gao Q., Tutt A., Zvelebil M., Ordonez L.D., Quist J., Tan D.W.-M., Isacke C.M., Grigoriadis A. (2015). Mouse mammary stem cells express prognostic markers for triple-negative breast cancer. Breast Cancer Res..

[7] Tiede B., Kang Y. (2011). From milk to malignancy: The role of mammary stem cells in development, pregnancy and breast cancer. Cell Res..

[8] Al-Hajj M., Wicha M.S., Benito-Hernandez A., Morrison S.J., Clarke M.F. (2003). Prospective identification of tumorigenic breast cancer cells. Proc. Natl. Acad. Sci. USA.

[9] Nami B., Ghasemi-Dizgah A., Vaseghi A. (2016). Overexpression of molecular chaperons GRP78 and GRP94 in CD44hi/CD24lo breast cancer stem cells. Bioimpacts.

[10] Doherty M.R., Smigiel J.M., Junk D.J., Jackson M.W. (2016). Cancer Stem Cell Plasticity Drives Therapeutic Resistance. Cancers.

[11] Draffin J.E., McFarlane S., Hill A., Johnston P.G., Waugh D.J.J. (2004). CD44 potentiates the adherence of metastatic prostate and breast cancer cells to bone marrow endothelial cells. Cancer Res..

[12] Pham P.V., Phan N.L.C., Nguyen N.T., Truong N.H., Duong T.T., Le D.V., Truong K.D., Phan N.K. (2011). Differentiation of breast cancer stem cells by knockdown of CD44: Promising differentiation therapy. J. Transl. Med..

[13] Naor D., Sionov R.V., Ish-Shalom D. (1997). CD44: Structure, function, and association with the malignant process. Adv. Cancer Res..

[14] Gao A.C., Lou W., Dong J.T., Isaacs J.T. (1997). CD44 is a metastasis suppressor gene for prostatic cancer located on human chromosome 11p13. Cancer Res..

[15] Ponta H., Sherman L., Herrlich P.A. (2003). CD44: From adhesion molecules to signalling regulators. Nat. Rev. Mol. Cell Biol..

[16] Bourguignon L.Y., Zhu H., Chu A., Iida N., Zhang L., Hung M.C. (1997). Interaction between the adhesion receptor, CD44, and the oncogene product, p185HER2, promotes human ovarian tumor cell activation. J. Biol. Chem..

[17] Meran S., Luo D.D., Simpson R., Martin J., Wells A., Steadman R., Phillips A.O. (2011). Hyaluronan facilitates transforming growth factor-beta1-dependent proliferation via CD44 and epidermal growth factor receptor interaction. J. Biol. Chem..

[18] Cho S.H., Park Y.S., Kim H.J., Kim C.H., Lim S.W., Huh J.W., Lee J.H., Kim H.R. (2012). CD44 enhances the epithelial-mesenchymal transition in association with colon cancer invasion. Int. J. Oncol..

[19] Marotta L.L.C., Almendro V., Marusyk A., Shipitsin M., Schemme J., Walker S.R., Bloushtain-Qimron N., Kim J.J., Choudhury S.A., Maruyama R. (2011). The JAK2/STAT3 signaling pathway is required for growth of CD44(+)CD24(−) stem cell-like breast cancer cells in human tumors. J. Clin. Investig..

[20] Baumann P., Cremers N., Kroese F., Orend G., Chiquet-Ehrismann R., Uede T., Yagita H., Sleeman J.P. (2005). CD24 expression causes the acquisition of multiple cellular properties associated with tumor growth and metastasis. Cancer Res..

[21] Aigner S., Sthoeger Z.M., Fogel M., Weber E., Zarn J., Ruppert M., Zeller Y., Vestweber D., Stahel R., Sammar M. (1997). CD24, a mucin-type glycoprotein, is a ligand for P-selectin on human tumor cells. Blood.

[22] Friederichs J., Zeller Y., Hafezi-Moghadam A., Grone H.J., Ley K., Altevogt P. (2000). The CD24/P-selectin binding pathway initiates lung arrest of human A125 adenocarcinoma cells. Cancer Res..

[23] Schabath H., Runz S., Joumaa S., Altevogt P. (2006). CD24 affects CXCR4 function in pre-B lymphocytes and breast carcinoma cells. J. Cell Sci..

[24] Ju J., Jang K., Lee K.-M., Kim M., Kim J., Yi J.Y., Noh D.-Y., Shin I. (2011). CD24 enhances DNA damage-induced apoptosis by modulating NF-kappaB signaling in CD44-expressing breast cancer cells. Carcinogenesis.

[25] Bretz N., Noske A., Keller S., Erbe-Hofmann N., Schlange T., Salnikov A.V., Moldenhauer G., Kristiansen G., Altevogt P. (2012). CD24 promotes tumor cell invasion by suppressing tissue factor pathway inhibitor-2 (TFPI-2) in a c-Src-dependent fashion. Clin. Exp. Metastasis.

[26] Ginestier C., Hur M.H., Charafe-Jauffret E., Monville F., Dutcher J., Brown M., Jacquemier J., Viens P., Kleer C., Liu S. (2007). ALDH1 is a marker of normal and malignant human mammary stem cells and a predictor of poor clinical outcome. Cell Stem Cell.

[27] Jackson B., Brocker C., Thompson D.C., Black W., Vasiliou K., Nebert D.W., Vasiliou V. (2011). Update on the aldehyde dehydrogenase gene (ALDH) superfamily. Hum. Genom..

[28] Hsu L.C., Chang W.C., Yoshida A. (1989). Genomic structure of the human cytosolic aldehyde dehydrogenase gene. Genomics.

[29] Thomas M., Halsall S., Peters T.J. (1982). Role of hepatic acetaldehyde dehydrogenase in alcoholism: Demonstration of persistent reduction of cytosolic activity in abstaining patients. Lancet.

[30] Tomita H., Tanaka K., Tanaka T., Hara A. (2016). Aldehyde dehydrogenase 1A1 in stem cells and cancer. Oncotarget.

[31] Ma I., Allan A.L. (2011). The role of human aldehyde dehydrogenase in normal and cancer stem cells. Stem Cell Rev..

[32] Honeth G., Bendahl P.-O., Ringner M., Saal L.H., Gruvberger-Saal S.K., Lovgren K., Grabau D., Ferno M., Borg A., Hegardt C. (2008). The CD44+/CD24− phenotype is enriched in basal-like breast tumors. Breast Cancer Res..

[33] Abraham B.K., Fritz P., McClellan M., Hauptvogel P., Athelogou M., Brauch H. (2005). Prevalence of CD44+/CD24−/low cells in breast cancer may not be associated with clinical outcome but may favor distant metastasis. Clin. Cancer Res..

[34] Liu H., Patel M.R., Prescher J.A., Patsialou A., Qian D., Lin J., Wen S., Chang Y.-F., Bachmann M.H., Shimono Y. (2010). Cancer stem cells from human breast tumors are involved in spontaneous metastases in orthotopic mouse models. Proc. Natl. Acad. Sci. USA.

[35] Aktas B., Tewes M., Fehm T., Hauch S., Kimmig R., Kasimir-Bauer S. (2009). Stem cell and epithelial-mesenchymal transition markers are frequently overexpressed in circulating tumor cells of metastatic breast cancer patients. Breast Cancer Res..

[36] Balic M., Lin H., Young L., Hawes D., Giuliano A., McNamara G., Datar R.H., Cote R.J. (2006). Most early disseminated cancer cells detected in bone marrow of breast cancer patients have a putative breast cancer stem cell phenotype. Clin. Cancer Res..

[37] Theodoropoulos P.A., Polioudaki H., Agelaki S., Kallergi G., Saridaki Z., Mavroudis D., Georgoulias V. (2010). Circulating tumor cells with a putative stem cell phenotype in peripheral blood of patients with breast cancer. Cancer Lett..

[38] Nami B., Donmez H., Kocak N. (2016). Tunicamycin-induced endoplasmic reticulum stress reduces in vitro subpopulation and invasion of CD44+/CD24− phenotype breast cancer stem cells. Exp. Toxicol. Pathol..

[39] Opyrchal M., Salisbury J.L., Iankov I., Goetz M.P., McCubrey J., Gambino M.W., Malatino L., Puccia G., Ingle J.N., Galanis E. (2014). Inhibition of Cdk2 kinase activity selectively targets the CD44(+)/CD24(−)/Low stem-like subpopulation and restores chemosensitivity of SUM149PT triple-negative breast cancer cells. Int. J. Oncol..

[40] Li X., Lewis M.T., Huang J., Gutierrez C., Osborne C.K., Wu M.-F., Hilsenbeck S.G., Pavlick A., Zhang X., Chamness G.C. (2008). Intrinsic resistance of tumorigenic breast cancer cells to chemotherapy. J. Natl. Cancer Inst..

[41] Creighton C.J., Li X., Landis M., Dixon J.M., Neumeister V.M., Sjolund A., Rimm D.L., Wong H., Rodriguez A., Herschkowitz J.I. (2009). Residual breast cancers after conventional therapy display mesenchymal as well as tumor-initiating features. Proc. Natl. Acad. Sci. USA.

[42] Croker A.K., Goodale D., Chu J., Postenka C., Hedley B.D., Hess D.A., Allan A.L. (2009). High aldehyde dehydrogenase and expression of cancer stem cell markers selects for breast cancer cells with enhanced malignant and metastatic ability. J. Cell. Mol. Med..

[43] Muleris M., Almeida A., Malfoy B., Dutrillaux B. (1997). Assignment of v-erb-b2 avian erythroblastic leukemia viral oncogene homolog 2 (ERBB2) to human chromosome band 17q21.1 by in situ hybridization. Cytogenet. Cell Genet..

[44] Coussens L., Yang-Feng T.L., Liao Y.C., Chen E., Gray A., McGrath J., Seeburg P.H., Libermann T.A., Schlessinger J., Francke U. (1985). Tyrosine kinase receptor with extensive homology to EGF receptor shares chromosomal location with neu oncogene. Science.

[45] Akiyama T., Sudo C., Ogawara H., Toyoshima K., Yamamoto T. (1986). The product of the human c-erbB-2 gene: A 185-kilodalton glycoprotein with tyrosine kinase activity. Science.

[46] Graus-Porta D., Beerli R.R., Daly J.M., Hynes N.E. (1997). ErbB-2, the preferred heterodimerization partner of all ErbB receptors, is a mediator of lateral signaling. EMBO J..

[47] King C.R., Borrello I., Porter L., Comoglio P., Schlessinger J. (1989). Ligand-independent tyrosine phosphorylation of EGF receptor and the erbB-2/neu proto-oncogene product is induced by hyperosmotic shock. Oncogene.

[48] Guo W., Pylayeva Y., Pepe A., Yoshioka T., Muller W.J., Inghirami G., Giancotti F.G. (2006). Beta 4 integrin amplifies ErbB2 signaling to promote mammary tumorigenesis. Cell.

[49] Lenferink A.E., Busse D., Flanagan W.M., Yakes F.M., Arteaga C.L. (2001). ErbB2/neu kinase modulates cellular p27(Kip1) and cyclin D1 through multiple signaling pathways. Cancer Res..

[50] Nelson J.M., Fry D.W. (2001). Akt, MAPK (Erk1/2), and p38 act in concert to promote apoptosis in response to ErbB receptor family inhibition. J. Biol. Chem..

[51] Li L.-Y., Chen H., Hsieh Y.-H., Wang Y.-N., Chu H.-J., Chen Y.-H., Chen H.-Y., Chien P.-J., Ma H.-T., Tsai H.-C. (2011). Nuclear ErbB2 enhances translation and cell growth by activating transcription of ribosomal RNA genes. Cancer Res..

[52] Wang S.-C., Lien H.-C., Xia W., Chen I.-F., Lo H.-W., Wang Z., Ali-Seyed M., Lee D.-F., Bartholomeusz G., Ou-Yang F. (2004). Binding at and transactivation of the COX-2 promoter by nuclear tyrosine kinase receptor ErbB-2. Cancer Cell.

[53] Chen Q.Q., Chen X.Y., Jiang Y.Y., Liu J. (2005). Identification of novel nuclear localization signal within the ErbB-2 protein. Cell Res..

[54] Maher M. (2014). Current and Emerging Treatment Regimens for HER2-Positive Breast Cancer. Pharm. Ther..

[55] Korkaya H., Paulson A., Iovino F., Wicha M.S. (2008). HER2 regulates the mammary stem/progenitor cell population driving tumorigenesis and invasion. Oncogene.

[56] Ithimakin S., Day K.C., Malik F., Zen Q., Dawsey S.J., Bersano-Begey T.F., Quraishi A.A., Ignatoski K.W., Daignault S., Davis A. (2013). HER2 drives luminal breast cancer stem cells in the absence of HER2 amplification: Implications for efficacy of adjuvant trastuzumab. Cancer Res..

[57] Gong C., Yao H., Liu Q., Chen J., Shi J., Su F., Song E. (2010). Markers of tumor-initiating cells predict chemoresistance in breast cancer. PLoS ONE.

[58] Liu J.C., Voisin V., Bader G.D., Deng T., Pusztai L., Symmans W.F., Esteva F.J., Egan S.E., Zacksenhaus E. (2012). Seventeen-gene signature from enriched Her2/Neu mammary tumor-initiating cells predicts clinical outcome for human HER2(+):ERα(−) breast cancer. Proc. Natl. Acad. Sci. USA.

[59] Magnifico A., Albano L., Campaner S., Delia D., Castiglioni F., Gasparini P., Sozzi G., Fontanella E., Menard S., Tagliabue E. (2009). Tumor-initiating cells of HER2-positive carcinoma cell lines express the highest oncoprotein levels and are sensitive to trastuzumab. Clin. Cancer Res..

[60] Son M.-Y., Kim J., Han H.-W., Woo S.-M., Cho Y.S., Kang Y.-K., Han Y.-M. (2008). Expression profiles of protein tyrosine kinase genes in human embryonic stem cells. Reproduction.

[61] Wang L., Schulz T.C., Sherrer E.S., Dauphin D.S., Shin S., Nelson A.M., Ware C.B., Zhan M., Song C.-Z., Chen X. (2007). Self-renewal of human embryonic stem cells requires insulin-like growth factor-1 receptor and ERBB2 receptor signaling. Blood.

[62] Zhou Y., Li S., Hu Y.P., Wang J., Hauser J., Conway A.N., Vinci M.A., Humphrey L., Zborowska E., Willson J.K.V. (2006). Blockade of EGFR and ErbB2 by the novel dual EGFR and ErbB2 tyrosine kinase inhibitor GW572016 sensitizes human colon carcinoma GEO cells to apoptosis. Cancer Res..

[63] Pivot X., Manikhas A., Zurawski B., Chmielowska E., Karaszewska B., Allerton R., Chan S., Fabi A., Bidoli P., Gori S. (2015). CEREBEL (EGF111438): A Phase III, Randomized, Open-label study of lapatinib plus capecitabine versus trastuzumab plus capecitabine in patients with human epidermal growth factor receptor 2-positive metastatic breast Cancer. J. Clin. Oncol..

[64] Chen Z.-L., Shen Y.-W., Li S.-T., Li C.-L., Zhang L.-X., Yang J., Lv M., Lin Y.-Y., Wang X., Yang J. (2016). The efficiency and safety of trastuzumab and lapatinib added to neoadjuvant chemotherapy in Her2-positive breast cancer patients: A randomized meta-analysis. Onco Targets Ther..

[65] Saleem A., Searle G.E., Kenny L.M., Huiban M., Kozlowski K., Waldman A.D., Woodley L., Palmieri C., Lowdell C., Kaneko T. (2015). Lapatinib access into normal brain and brain metastases in patients with Her-2 overexpressing breast cancer. EJNMMI Res..

[66] Lee C.Y.-F., Lin Y., Bratman S.V., Feng W., Kuo A.H., Scheeren F.A., Engreitz J.M., Varma S., West R.B., Diehn M. (2014). Neuregulin autocrine signaling promotes self-renewal of breast tumor-initiating cells by triggering HER2/HER3 activation. Cancer Res..

[67] Farnie G., Johnson R.L., Williams K.E., Clarke R.B., Bundred N.J. (2014). Lapatinib inhibits stem/progenitor proliferation in preclinical in vitro models of ductal carcinoma in situ (DCIS). Cell Cycle.

[68] Ohnishi Y., Yasui H., Kakudo K., Nozaki M. (2016). Lapatinib-resistant cancer cells possessing epithelial cancer stem cell properties develop sensitivity during sphere formation by activation of the ErbB/AKT/cyclin D2 pathway. Oncol. Rep..

[69] Liu J., Chen X., Mao Y., Qu Q., Shen K. (2014). Association of epithelial-mesenchymal transition with lapatinib resistance through multipe pathways activation in HER2-positive breast cancer. J. Clin. Oncol..

[70] Carter P., Presta L., Gorman C.M., Ridgway J.B., Henner D., Wong W.L., Rowland A.M., Kotts C., Carver M.E., Shepard H.M. (1992). Humanization of an anti-p185HER2 antibody for human cancer therapy. Proc. Natl. Acad. Sci. USA.

[71] Fendly B.M., Winget M., Hudziak R.M., Lipari M.T., Napier M.A., Ullrich A. (1990). Characterization of murine monoclonal antibodies reactive to either the human epidermal growth factor receptor or HER2/neu gene product. Cancer Res..

[72] Junttila T.T., Akita R.W., Parsons K., Fields C., Lewis Phillips G.D., Friedman L.S., Sampath D., Sliwkowski M.X. (2009). Ligand-independent HER2/HER3/PI3K complex is disrupted by trastuzumab and is effectively inhibited by the PI3K inhibitor GDC-0941. Cancer Cell.

[73] Nagata Y., Lan K.-H., Zhou X., Tan M., Esteva F.J., Sahin A.A., Klos K.S., Li P., Monia B.P., Nguyen N.T. (2004). PTEN activation contributes to tumor inhibition by trastuzumab, and loss of PTEN predicts trastuzumab resistance in patients. Cancer Cell.

[74] Zhang S., Huang W.-C., Li P., Guo H., Poh S.-B., Brady S.W., Xiong Y., Tseng L.-M., Li S.-H., Ding Z. (2011). Combating trastuzumab resistance by targeting SRC, a common node downstream of multiple resistance pathways. Nat. Med..

[75] Nahta R., Takahashi T., Ueno N.T., Hung M.-C., Esteva F.J. (2004). P27(kip1) down-regulation is associated with trastuzumab resistance in breast cancer cells. Cancer Res..

[76] Le X.-F., Bedrosian I., Mao W., Murray M., Lu Z., Keyomarsi K., Lee M.-H., Zhao J., Bast R.C.J. (2006). Anti-HER2 antibody trastuzumab inhibits CDK2-mediated NPAT and histone H4 expression via the PI3K pathway. Cell Cycle.

[77] Scaltriti M., Eichhorn P.J., Cortes J., Prudkin L., Aura C., Jimenez J., Chandarlapaty S., Serra V., Prat A., Ibrahim Y.H. (2011). Cyclin E amplification/overexpression is a mechanism of trastuzumab resistance in HER2+ breast cancer patients. Proc. Natl. Acad. Sci. USA.

[78] Klapper L.N., Waterman H., Sela M., Yarden Y. (2000). Tumor-inhibitory antibodies to HER-2/ErbB-2 may act by recruiting c-Cbl and enhancing ubiquitination of HER-2. Cancer Res..

[79] Clynes R.A., Towers T.L., Presta L.G., Ravetch J.V. (2000). Inhibitory Fc receptors modulate in vivo cytotoxicity against tumor targets. Nat. Med..

[80] Raab S., Steinbacher J., Schmiedel B.J., Kousis P.C., Steinle A., Jung G., Grosse-Hovest L., Salih H.R. (2014). Fc-optimized NKG2D-Fc constructs induce NK cell antibody-dependent cellular cytotoxicity against breast cancer cells independently of HER2/neu expression status. J. Immunol..

[81] Esteva F.J., Valero V., Booser D., Guerra L.T., Murray J.L., Pusztai L., Cristofanilli M., Arun B., Esmaeli B., Fritsche H.A. (2002). Phase II study of weekly docetaxel and trastuzumab for patients with HER-2-overexpressing metastatic breast cancer. J. Clin. Oncol..

[82] Sato N., Sano M., Tabei T., Asaga T., Ando J., Fujii H., Yamamoto N., Kurosumi M., Inoue K., Kimura M. (2006). Combination docetaxel and trastuzumab treatment for patients with HER-2-overexpressing metastatic breast cancer: A multicenter, phase-II study. Breast Cancer.

[83] Marty M., Cognetti F., Maraninchi D., Snyder R., Mauriac L., Tubiana-Hulin M., Chan S., Grimes D., Anton A., Lluch A. (2005). Randomized phase II trial of the efficacy and safety of trastuzumab combined with docetaxel in patients with human epidermal growth factor receptor 2-positive metastatic breast cancer administered as first-line treatment: The M77001 study group. J. Clin. Oncol..

[84] Gianni L., Pienkowski T., Im Y.-H., Roman L., Tseng L.-M., Liu M.-C., Lluch A., Staroslawska E., de la Haba-Rodriguez J., Im S.-A. (2012). Efficacy and safety of neoadjuvant pertuzumab and trastuzumab in women with locally advanced, inflammatory, or early HER2-positive breast cancer (NeoSphere): A randomised multicentre, open-label, phase 2 trial. Lancet. Oncol..

[85] Slamon D.J., Leyland-Jones B., Shak S., Fuchs H., Paton V., Bajamonde A., Fleming T., Eiermann W., Wolter J., Pegram M. (2001). Use of chemotherapy plus a monoclonal antibody against HER2 for metastatic breast cancer that overexpresses HER2. N. Engl. J. Med..

[86] Vogel C.L., Cobleigh M.A., Tripathy D., Gutheil J.C., Harris L.N., Fehrenbacher L., Slamon D.J., Murphy M., Novotny W.F., Burchmore M. (2002). Efficacy and safety of trastuzumab as a single agent in first-line treatment of HER2-overexpressing metastatic breast cancer. J. Clin. Oncol..

[87] Eiermann W. (2001). Trastuzumab combined with chemotherapy for the treatment of HER2-positive metastatic breast cancer: Pivotal trial data. Ann. Oncol. Off. J. Eur. Soc. Med. Oncol..

[88] Dahabreh I.J., Linardou H., Siannis F., Fountzilas G., Murray S. (2008). Trastuzumab in the adjuvant treatment of early-stage breast cancer: A systematic review and meta-analysis of randomized controlled trials. Oncologist.

[89] Piccart-Gebhart M.J., Procter M., Leyland-Jones B., Goldhirsch A., Untch M., Smith I., Gianni L., Baselga J., Bell R., Jackisch C. (2005). Trastuzumab after adjuvant chemotherapy in HER2-positive breast cancer. N. Engl. J. Med..

[90] Kalluri R., Weinberg R.A. (2009). The basics of epithelial-mesenchymal transition. J. Clin. Investig..

[91] Lamouille S., Xu J., Derynck R. (2014). Molecular mechanisms of epithelial–mesenchymal transition. Nat. Rev. Mol. Cell Biol..

[92] Mani S.A., Guo W., Liao M.-J., Eaton E.N., Ayyanan A., Zhou A.Y., Brooks M., Reinhard F., Zhang C.C., Shipitsin M. (2008). The epithelial-mesenchymal transition generates cells with properties of stem cells. Cell.

[93] Giordano A., Mego M., Lee B., Anfossi S., Parker C.A., Alvarez R.H., Ueno N.T., Valero V., Cristofanilli M., Reuben J.M. (2011). Epithelial-mesenchymal transition in patients with HER2+ metastatic breast cancer. J. Clin. Oncol..

[94] Giordano A., Gao H., Anfossi S., Cohen E., Mego M., Lee B.-N., Tin S., De Laurentiis M., Parker C.A., Alvarez R.H. (2012). Epithelial-mesenchymal transition and stem cell markers in patients with HER2-positive metastatic breast cancer. Mol. Cancer Ther..

[95] Jenndahl L.E., Isakson P., Baeckstrom D. (2005). c-erbB2-induced epithelial-mesenchymal transition in mammary epithelial cells is suppressed by cell-cell contact and initiated prior to E-cadherin downregulation. Int. J. Oncol..

[96] Nilsson G.M.A., Akhtar N., Kannius-Janson M., Baeckstrom D. (2014). Loss of E-cadherin expression is not a prerequisite for c-ERBB2-induced epithelial-mesenchymal transition. Int. J. Oncol..

[97] Ingthorsson S., Andersen K., Hilmarsdottir B., Maelandsmo G.M., Magnusson M.K., Gudjonsson T. (2016). HER2 induced EMT and tumorigenicity in breast epithelial progenitor cells is inhibited by coexpression of EGFR. Oncogene.

[98] Rennstam K., Jonsson G., Tanner M., Bendahl P.-O., Staaf J., Kapanen A.I., Karhu R., Baldetorp B., Borg A., Isola J. (2007). Cytogenetic characterization and gene expression profiling of the trastuzumab-resistant breast cancer cell line JIMT-1. Cancer Genet. Cytogenet..

[99] Barok M., Isola J., Palyi-Krekk Z., Nagy P., Juhasz I., Vereb G., Kauraniemi P., Kapanen A., Tanner M., Vereb G. (2007). Trastuzumab causes antibody-dependent cellular cytotoxicity-mediated growth inhibition of submacroscopic JIMT-1 breast cancer xenografts despite intrinsic drug resistance. Mol. Cancer Ther..

[100] Koninki K., Barok M., Tanner M., Staff S., Pitkanen J., Hemmila P., Ilvesaro J., Isola J. (2010). Multiple molecular mechanisms underlying trastuzumab and lapatinib resistance in JIMT-1 breast cancer cells. Cancer Lett..

[101] Cavaliere C., Corvigno S., Galgani M., Limite G., Nardone A., Veneziani B.M. (2010). Combined inhibitory effect of formestane and herceptin on a subpopulation of CD44+/CD24low breast cancer cells. Cancer Sci..

[102] Oliveras-Ferraros C., Corominas-Faja B., Cufi S., Vazquez-Martin A., Martin-Castillo B., Iglesias J.M., Lopez-Bonet E., Martin A.G., Menendez J.A. (2012). Epithelial-to-mesenchymal transition (EMT) confers primary resistance to trastuzumab (Herceptin). Cell Cycle.

[103] Cufí S., Corominas-Faja B., Vazquez-Martin A., Oliveras-Ferraros C., Dorca J., Bosch-Barrera J., Martin-Castillo B., Menendez J.A. (2012). Metformin-induced preferential killing of breast cancer initiating CD44(+)CD24(−/low) cells is sufficient to overcome primary resistance to trastuzumab in HER2+ human breast cancer xenografts. Oncotarget.

[104] Oliveras-Ferraros C., Vazquez-Martin A., Martin-Castillo B., Cufi S., Del Barco S., Lopez-Bonet E., Brunet J., Menendez J.A. (2010). Dynamic emergence of the mesenchymal CD44(pos)CD24(neg/low) phenotype in HER2-gene amplified breast cancer cells with de novo resistance to trastuzumab (Herceptin). Biochem. Biophys. Res. Commun..

[105] Bauerschmitz G.J., Ranki T., Kangasniemi L., Ribacka C., Eriksson M., Porten M., Herrmann I., Ristimaki A., Virkkunen P., Tarkkanen M. (2008). Tissue-specific promoters active in CD44+CD24-/low breast cancer cells. Cancer Res..

[106] Rennstam K., McMichael N., Berglund P., Honeth G., Hegardt C., Ryden L., Luts L., Bendahl P.-O., Hedenfalk I. (2010). Numb protein expression correlates with a basal-like phenotype and cancer stem cell markers in primary breast cancer. Breast Cancer Res. Treat..

[107] Lesniak D., Sabri S., Xu Y., Graham K., Bhatnagar P., Suresh M., Abdulkarim B. (2013). Spontaneous Epithelial-Mesenchymal Transition and Resistance to HER-2-Targeted Therapies in HER-2-Positive Luminal Breast Cancer. PLoS ONE.

[108] Reim F., Dombrowski Y., Ritter C., Buttmann M., Hausler S., Ossadnik M., Krockenberger M., Beier D., Beier C.P., Dietl J. (2009). Immunoselection of breast and ovarian cancer cells with trastuzumab and natural killer cells: Selective escape of CD44high/CD24low/HER2low breast cancer stem cells. Cancer Res..

[109] Scaltriti M., Rojo F., Ocana A., Anido J., Guzman M., Cortes J., Di Cosimo S., Matias-Guiu X., Ramon y Cajal S., Arribas J. (2007). Expression of p95HER2, a truncated form of the HER2 receptor, and response to anti-HER2 therapies in breast cancer. J. Natl. Cancer Inst..

[110] Sperinde J., Jin X., Banerjee J., Penuel E., Saha A., Diedrich G., Huang W., Leitzel K., Weidler J., Ali S.M. (2010). Quantitation of p95HER2 in paraffin sections by using a p95-specific antibody and correlation with outcome in a cohort of trastuzumab-treated breast cancer patients. Clin. Cancer Res..

[111] Arribas J., Baselga J., Pedersen K., Parra-Palau J.L. (2011). p95HER2 and breast cancer. Cancer Res..

[112] Codony-Servat J., Albanell J., Lopez-Talavera J.C., Arribas J., Baselga J. (1999). Cleavage of the HER2 ectodomain is a pervanadate-activable process that is inhibited by the tissue inhibitor of metalloproteases-1 in breast cancer cells. Cancer Res..

[113] Yuan C.-X., Lasut A.L., Wynn R., Neff N.T., Hollis G.F., Ramaker M.L., Rupar M.J., Liu P., Meade R. (2003). Purification of Her-2 extracellular domain and identification of its cleavage site. Protein Expr. Purif..

[114] Sanderson M.P., Dempsey P.J., Dunbar A.J. (2006). Control of ErbB signaling through metalloprotease mediated ectodomain shedding of EGF-like factors. Growth Factors.

[115] Pedersen K., Angelini P.-D., Laos S., Bach-Faig A., Cunningham M.P., Ferrer-Ramon C., Luque-Garcia A., Garcia-Castillo J., Parra-Palau J.L., Scaltriti M. (2009). A naturally occurring HER2 carboxy-terminal fragment promotes mammary tumor growth and metastasis. Mol. Cell. Biol..

[116] Ruff M., Leyme A., Le Cann F., Bonnier D., Le Seyec J., Chesnel F., Fattet L., Rimokh R., Baffet G., Theret N. (2015). The disintegrin and metalloprotease ADAM12 is associated with TGF-beta-induced epithelial to mesenchymal transition. PLoS ONE.

[117] Duhachek-Muggy S., Qi Y., Wise R., Alyahya L., Li H., Hodge J., Zolkiewska A. (2017). Metalloprotease-disintegrin ADAM12 actively promotes the stem cell-like phenotype in claudin-low breast cancer. Mol. Cancer.

[118] Pulyaeva H., Bueno J., Polette M., Birembaut P., Sato H., Seiki M., Thompson E.W. (1997). MT1-MMP correlates with MMP-2 activation potential seen after epithelial to mesenchymal transition in human breast carcinoma cells. Clin. Exp. Metastasis.

[119] Tester A.M., Ruangpanit N., Anderson R.L., Thompson E.W. (2000). MMP-9 secretion and MMP-2 activation distinguish invasive and metastatic sublines of a mouse mammary carcinoma system showing epithelial-mesenchymal transition traits. Clin. Exp. Metastasis.

[120] O’Shea C., McKie N., Buggy Y., Duggan C., Hill A.D.K., McDermott E., O’Higgins N., Duffy M.J. (2003). Expression of ADAM-9 mRNA and protein in human breast cancer. Int. J. Cancer.

[121] Roy R., Wewer U.M., Zurakowski D., Pories S.E., Moses M.A. (2004). ADAM 12 cleaves extracellular matrix proteins and correlates with cancer status and stage. J. Biol. Chem..

[122] Lendeckel U., Kohl J., Arndt M., Carl-McGrath S., Donat H., Rocken C. (2005). Increased expression of ADAM family members in human breast cancer and breast cancer cell lines. J. Cancer Res. Clin. Oncol..

[123] Mitsui Y., Mochizuki S., Kodama T., Shimoda M., Ohtsuka T., Shiomi T., Chijiiwa M., Ikeda T., Kitajima M., Okada Y. (2006). ADAM28 is overexpressed in human breast carcinomas: Implications for carcinoma cell proliferation through cleavage of insulin-like growth factor binding protein-3. Cancer Res..

[124] McGowan P.M., Ryan B.M., Hill A.D.K., McDermott E., O’Higgins N., Duffy M.J. (2007). ADAM-17 expression in breast cancer correlates with variables of tumor progression. Clin. Cancer Res..

[125] Mullooly M., McGowan P.M., Kennedy S.A., Madden S.F., Crown J., O’ Donovan N., Duffy M.J. (2015). ADAM10: A new player in breast cancer progression?. Br. J. Cancer.

[126] Köhrmann A., Kammerer U., Kapp M., Dietl J., Anacker J. (2009). Expression of matrix metalloproteinases (MMPs) in primary human breast cancer and breast cancer cell lines: New findings and review of the literature. BMC Cancer.

[127] Benson C.S., Babu S.D., Radhakrishna S., Selvamurugan N., Ravi Sankar B. (2013). Expression of matrix metalloproteinases in human breast cancer tissues. Dis. Markers.

[128] Bartsch J.E., Staren E.D., Appert H.E. (2003). Matrix metalloproteinase expression in breast cancer. J. Surg. Res..

[129] Merdad A., Karim S., Schulten H.-J., Dallol A., Buhmeida A., Al-Thubaity F., Gari M.A., Chaudhary A.G., Abuzenadah A.M., Al-Qahtani M.H. (2014). Expression of matrix metalloproteinases (MMPs) in primary human breast cancer: MMP-9 as a potential biomarker for cancer invasion and metastasis. Anticancer Res..

[130] Liu P.C.C., Liu X., Li Y., Covington M., Wynn R., Huber R., Hillman M., Yang G., Ellis D., Marando C. (2006). Identification of ADAM10 as a major source of HER2 ectodomain sheddase activity in HER2 overexpressing breast cancer cells. Cancer Biol. Ther..

[131] Feldinger K., Generali D., Kramer-Marek G., Gijsen M., Ng T.B., Wong J.H., Strina C., Cappelletti M., Andreis D., Li J.-L. (2014). ADAM10 mediates trastuzumab resistance and is correlated with survival in HER2 positive breast cancer. Oncotarget.

[132] Gijsen M., King P., Perera T., Parker P.J., Harris A.L., Larijani B., Kong A. (2010). HER2 phosphorylation is maintained by a PKB negative feedback loop in response to anti-HER2 herceptin in breast cancer. PLoS Biol..

[133] Caiazza F., McGowan P.M., Mullooly M., Murray A., Synnott N., O’Donovan N., Flanagan L., Tape C.J., Murphy G., Crown J. (2015). Targeting ADAM-17 with an inhibitory monoclonal antibody has antitumour effects in triple-negative breast cancer cells. Br. J. Cancer.

[134] Duffy M.J., Mullooly M., O’Donovan N., Sukor S., Crown J., Pierce A., McGowan P.M. (2011). The ADAMs family of proteases: New biomarkers and therapeutic targets for cancer?. Clin. Proteomics.

[135] Burns D.M., He C., Li Y., Scherle P., Liu X., Marando C.A., Covington M.B., Yang G., Pan M., Turner S. (2008). Conversion of an MMP-potent scaffold to an MMP-selective HER-2 sheddase inhibitor via scaffold hybridization and subtle P1’ permutations. Bioorg. Med. Chem. Lett..

[136] Duchnowska R., Sperinde J., Chenna A., Haddad M., Paquet A., Lie Y., Weidler J.M., Huang W., Winslow J., Jankowski T. (2014). Quantitative measurements of tumoral p95HER2 protein expression in metastatic breast cancer patients treated with trastuzumab: Independent validation of the p95HER2 clinical cutoff. Clin. Cancer Res..

[137] Han S.-W., Cha Y., Paquet A., Huang W., Weidler J., Lie Y., Sherwood T., Bates M., Haddad M., Park I.H. (2012). Correlation of HER2, p95HER2 and HER3 expression and treatment outcome of lapatinib plus capecitabine in her2-positive metastatic breast cancer. PLoS ONE.

[138] Lipton A., Goodman L., Leitzel K., Cook J., Sperinde J., Haddad M., Kostler W.J., Huang W., Weidler J.M., Ali S. (2013). HER3, p95HER2, and HER2 protein expression levels define multiple subtypes of HER2-positive metastatic breast cancer. Breast Cancer Res. Treat..

[139] Saez R., Molina M.A., Ramsey E.E., Rojo F., Keenan E.J., Albanell J., Lluch A., Garcia-Conde J., Baselga J., Clinton G.M. (2006). p95HER-2 predicts worse outcome in patients with HER-2-positive breast cancer. Clin. Cancer Res..

[140] Watabe T., Miyazono K. (2009). Roles of TGF-beta family signaling in stem cell renewal and differentiation. Cell Res..

[141] Derynck R., Rhee L., Chen E.Y., Van Tilburg A. (1987). Intron-exon structure of the human transforming growth factor-beta precursor gene. Nucleic Acids Res..

[142] Dickinson M.E., Kobrin M.S., Silan C.M., Kingsley D.M., Justice M.J., Miller D.A., Ceci J.D., Lock L.F., Lee A., Buchberg A.M. (1990). Chromosomal localization of seven members of the murine TGF-beta superfamily suggests close linkage to several morphogenetic mutant loci. Genomics.

[143] Wrana J.L., Attisano L., Carcamo J., Zentella A., Doody J., Laiho M., Wang X.F., Massague J. (1992). TGF beta signals through a heteromeric protein kinase receptor complex. Cell.

[144] Yamashita H., ten Dijke P., Franzen P., Miyazono K., Heldin C.H. (1994). Formation of hetero-oligomeric complexes of type I and type II receptors for transforming growth factor-beta. J. Biol. Chem..

[145] Massague J. (2012). TGFbeta signalling in context. Nat. Rev. Mol. Cell Biol..

[146] Zhang Y.E. (2009). Non-Smad pathways in TGF-beta signaling. Cell Res..

[147] Kim H., Choi J.-A., Kim J.-H. (2014). Ras promotes transforming growth factor-beta (TGF-beta)-induced epithelial-mesenchymal transition via a leukotriene B4 receptor-2-linked cascade in mammary epithelial cells. J. Biol. Chem..

[148] Mori S., Kodaira M., Ito A., Okazaki M., Kawaguchi N., Hamada Y., Takada Y., Matsuura N. (2015). Enhanced expression of integrin alphavbeta3 induced by TGF-beta is required for the enhancing effect of fibroblast growth factor 1 (FGF1) in TGF-beta-induced epithelial-mesenchymal transition (EMT) in mammary epithelial cells. PLoS ONE.

[149] Pang M.-F., Georgoudaki A.-M., Lambut L., Johansson J., Tabor V., Hagikura K., Jin Y., Jansson M., Alexander J.S., Nelson C.M. (2016). TGF-beta1-induced EMT promotes targeted migration of breast cancer cells through the lymphatic system by the activation of CCR7/CCL21-mediated chemotaxis. Oncogene.

[150] Asiedu M.K., Ingle J.N., Behrens M.D., Radisky D.C., Knutson K.L. (2011). TGFβ/TNFα-nediated epithelial-mesenchymal transition generates breast cancer stem cells with a claudin-low phenotype. Cancer Res..

[151] Stankic M., Pavlovic S., Chin Y., Brogi E., Padua D., Norton L., Massague J., Benezra R. (2013). TGF-beta-Id1 signaling opposes Twist1 and promotes metastatic colonization via a mesenchymal-to-epithelial transition. Cell Rep..

[152] Shirakihara T., Saitoh M., Miyazono K. (2007). Differential regulation of epithelial and mesenchymal markers by deltaEF1 proteins in epithelial mesenchymal transition induced by TGF-beta. Mol. Biol. Cell.

[153] Cano A., Perez-Moreno M.A., Rodrigo I., Locascio A., Blanco M.J., del Barrio M.G., Portillo F., Nieto M.A. (2000). The transcription factor snail controls epithelial-mesenchymal transitions by repressing E-cadherin expression. Nat. Cell Biol..

[154] Savagner P., Yamada K.M., Thiery J.P. (1997). The zinc-finger protein slug causes desmosome dissociation, an initial and necessary step for growth factor-induced epithelial-mesenchymal transition. J. Cell Biol..

[155] Comijn J., Berx G., Vermassen P., Verschueren K., van Grunsven L., Bruyneel E., Mareel M., Huylebroeck D., van Roy F. (2001). The two-handed E box binding zinc finger protein SIP1 downregulates E-cadherin and induces invasion. Mol. Cell.

[156] Thuault S., Valcourt U., Petersen M., Manfioletti G., Heldin C.-H., Moustakas A. (2006). Transforming growth factor-beta employs HMGA2 to elicit epithelial-mesenchymal transition. J. Cell Biol..

[157] Koinuma D., Tsutsumi S., Kamimura N., Taniguchi H., Miyazawa K., Sunamura M., Imamura T., Miyazono K., Aburatani H. (2009). Chromatin immunoprecipitation on microarray analysis of Smad2/3 binding sites reveals roles of ETS1 and TFAP2A in transforming growth factor beta signaling. Mol. Cell. Biol..

[158] Kowanetz M., Valcourt U., Bergstrom R., Heldin C.-H., Moustakas A. (2004). Id2 and Id3 define the potency of cell proliferation and differentiation responses to transforming growth factor beta and bone morphogenetic protein. Mol. Cell. Biol..

[159] Kondo M., Cubillo E., Tobiume K., Shirakihara T., Fukuda N., Suzuki H., Shimizu K., Takehara K., Cano A., Saitoh M. (2004). A role for Id in the regulation of TGF-beta-induced epithelial-mesenchymal transdifferentiation. Cell Death Differ..

[160] Papageorgis P., Lambert A.W., Ozturk S., Gao F., Pan H., Manne U., Alekseyev Y.O., Thiagalingam A., Abdolmaleky H.M., Lenburg M. (2010). Smad signaling is required to maintain epigenetic silencing during breast cancer progression. Cancer Res..

[161] Gregory P.A., Bert A.G., Paterson E.L., Barry S.C., Tsykin A., Farshid G., Vadas M.A., Khew-Goodall Y., Goodall G.J. (2008). The miR-200 family and miR-205 regulate epithelial to mesenchymal transition by targeting ZEB1 and SIP1. Nat. Cell Biol..

[162] Park S.-M., Gaur A.B., Lengyel E., Peter M.E. (2008). The miR-200 family determines the epithelial phenotype of cancer cells by targeting the E-cadherin repressors ZEB1 and ZEB. Genes Dev..

[163] Korpal M., Lee E.S., Hu G., Kang Y. (2008). The miR-200 family inhibits epithelial-mesenchymal transition and cancer cell migration by direct targeting of E-cadherin transcriptional repressors ZEB1 and ZEB2. J. Biol. Chem..

[164] Burk U., Schubert J., Wellner U., Schmalhofer O., Vincan E., Spaderna S., Brabletz T. (2008). A reciprocal repression between ZEB1 and members of the miR-200 family promotes EMT and invasion in cancer cells. EMBO Rep..

[165] Morita T., Mayanagi T., Sobue K. (2007). Dual roles of myocardin-related transcription factors in epithelial mesenchymal transition via slug induction and actin remodeling. J. Cell Biol..

[166] Yu L., Hebert M.C., Zhang Y.E. (2002). TGF-beta receptor-activated p38 MAP kinase mediates Smad-independent TGF-beta responses. EMBO J..

[167] Papageorgis P. (2015). TGFβ signaling in tumor initiation, epithelial-to-mesenchymal transition, and metastasis. J. Oncol..

[168] Lamouille S., Derynck R. (2007). Cell size and invasion in TGF-beta-induced epithelial to mesenchymal transition is regulated by activation of the mTOR pathway. J. Cell Biol..

[169] Lamouille S., Connolly E., Smyth J.W., Akhurst R.J., Derynck R. (2012). TGF-beta-induced activation of mTOR complex 2 drives epithelial-mesenchymal transition and cell invasion. J. Cell Sci..

[170] Bakin A.V., Tomlinson A.K., Bhowmick N.A., Moses H.L., Arteaga C.L. (2000). Phosphatidylinositol 3-kinase function is required for transforming growth factor beta-mediated epithelial to mesenchymal transition and cell migration. J. Biol. Chem..

[171] Zhou B.P., Deng J., Xia W., Xu J., Li Y.M., Gunduz M., Hung M.-C. (2004). Dual regulation of Snail by GSK-3beta-mediated phosphorylation in control of epithelial-mesenchymal transition. Nat. Cell Biol..

[172] Bachelder R.E., Yoon S.-O., Franci C., de Herreros A.G., Mercurio A.M. (2005). Glycogen synthase kinase-3 is an endogenous inhibitor of Snail transcription: Implications for the epithelial-mesenchymal transition. J. Cell Biol..

[173] Chaudhury A., Hussey G.S., Ray P.S., Jin G., Fox P.L., Howe P.H. (2010). TGF-beta-mediated phosphorylation of hnRNP E1 induces EMT via transcript-selective translational induction of Dab2 and ILEI. Nat. Cell Biol..

[174] Derynck R., Zhang Y.E. (2003). Smad-dependent and Smad-independent pathways in TGF-beta family signalling. Nature.

[175] Marchetti A., Colletti M., Cozzolino A.M., Steindler C., Lunadei M., Mancone C., Tripodi M. (2008). ERK5/MAPK is activated by TGFbeta in hepatocytes and required for the GSK-3beta-mediated Snail protein stabilization. Cell. Signal..

[176] Horiguchi K., Shirakihara T., Nakano A., Imamura T., Miyazono K., Saitoh M. (2009). Role of Ras signaling in the induction of snail by transforming growth factor-beta. J. Biol. Chem..

[177] Morel A.-P., Lievre M., Thomas C., Hinkal G., Ansieau S., Puisieux A. (2008). Generation of breast cancer stem cells through epithelial-mesenchymal transition. PLoS ONE.

[178] Scheel C., Eaton E.N., Li S.H.-J., Chaffer C.L., Reinhardt F., Kah K.-J., Bell G., Guo W., Rubin J., Richardson A.L. (2011). Paracrine and autocrine signals induce and maintain mesenchymal and stem cell states in the breast. Cell.

[179] Buijs J.T., van der Horst G., van den Hoogen C., Cheung H., de Rooij B., Kroon J., Petersen M., van Overveld P.G.M., Pelger R.C.M., van der Pluijm G. (2012). The BMP2/7 heterodimer inhibits the human breast cancer stem cell subpopulation and bone metastases formation. Oncogene.

[180] Santibanez J.F. (2006). JNK mediates TGF-beta1-induced epithelial mesenchymal transdifferentiation of mouse transformed keratinocytes. FEBS Lett..

[181] Tavares A.L.P., Mercado-Pimentel M.E., Runyan R.B., Kitten G.T. (2006). TGF beta-mediated RhoA expression is necessary for epithelial-mesenchymal transition in the embryonic chick heart. Dev. Dyn..

[182] Bhowmick N.A., Ghiassi M., Bakin A., Aakre M., Lundquist C.A., Engel M.E., Arteaga C.L., Moses H.L. (2001). Transforming growth factor-beta1 mediates epithelial to mesenchymal transdifferentiation through a RhoA-dependent mechanism. Mol. Biol. Cell.

[183] Wang S.E. (2011). The functional crosstalk between HER2 tyrosine kinase and TGF-β signaling in breast cancer malignancy. J. Signal Transduct..

[184] Ueda Y., Wang S., Dumont N., Yi J.Y., Koh Y., Arteaga C.L. (2004). Overexpression of HER2 (erbB2) in human breast epithelial cells unmasks transforming growth factor beta-induced cell motility. J. Biol. Chem..

[185] Siegel P.M., Shu W., Cardiff R.D., Muller W.J., Massague J. (2003). Transforming growth factor beta signaling impairs Neu-induced mammary tumorigenesis while promoting pulmonary metastasis. Proc. Natl. Acad. Sci. USA.

[186] Muraoka R.S., Koh Y., Roebuck L.R., Sanders M.E., Brantley-Sieders D., Gorska A.E., Moses H.L., Arteaga C.L. (2003). Increased malignancy of Neu-induced mammary tumors overexpressing active transforming growth factor beta1. Mol. Cell. Biol..

[187] Seton-Rogers S.E., Lu Y., Hines L.M., Koundinya M., LaBaer J., Muthuswamy S.K., Brugge J.S. (2004). Cooperation of the ErbB2 receptor and transforming growth factor beta in induction of migration and invasion in mammary epithelial cells. Proc. Natl. Acad. Sci. USA.

[188] Zheng L., Ren J.Q., Li H., Kong Z.L., Zhu H.G. (2004). Downregulation of wild-type p53 protein by HER-2/neu mediated PI3K pathway activation in human breast cancer cells: Its effect on cell proliferation and implication for therapy. Cell Res..

[189] Yu Y., Wang Y., Ren X., Tsuyada A., Li A., Liu L.J., Wang S.E. (2010). Context-dependent bidirectional regulation of the MutS homolog 2 by transforming growth factor beta contributes to chemoresistance in breast cancer cells. Mol. Cancer Res..

[190] Chow A., Arteaga C.L., Wang S.E. (2011). When tumor suppressor TGFβ meets the HER2 (ERBB2) oncogene. J. Mammary Gland Biol. Neoplasia.

[191] Baker A.T., Zlobin A., Osipo C. (2014). Notch-EGFR/HER2 bidirectional crosstalk in breast cancer. Front. Oncol..

[192] Rebay I., Fehon R.G., Artavanis-Tsakonas S. (1993). Specific truncations of Drosophila Notch define dominant activated and dominant negative forms of the receptor. Cell.

[193] Rebay I., Fleming R.J., Fehon R.G., Cherbas L., Cherbas P., Artavanis-Tsakonas S. (1991). Specific EGF repeats of Notch mediate interactions with Delta and Serrate: Implications for Notch as a multifunctional receptor. Cell.

[194] Fitzgerald K., Greenwald I. (1995). Interchangeability of Caenorhabditis elegans DSL proteins and intrinsic signalling activity of their extracellular domains in vivo. Development.

[195] Henderson S.T., Gao D., Christensen S., Kimble J. (1997). Functional domains of LAG-2, a putative signaling ligand for LIN-12 and GLP-1 receptors in Caenorhabditis elegans. Mol. Biol. Cell.

[196] Schroeter E.H., Kisslinger J.A., Kopan R. (1998). Notch-1 signalling requires ligand-induced proteolytic release of intracellular domain. Nature.

[197] Mumm J.S., Schroeter E.H., Saxena M.T., Griesemer A., Tian X., Pan D.J., Ray W.J., Kopan R. (2000). A ligand-induced extracellular cleavage regulates gamma-secretase-like proteolytic activation of Notch. Mol. Cell.

[198] Brou C., Logeat F., Gupta N., Bessia C., LeBail O., Doedens J.R., Cumano A., Roux P., Black R.A., Israel A. (2000). A novel proteolytic cleavage involved in Notch signaling: The role of the disintegrin-metalloprotease TACE. Mol. Cell.

[199] Dontu G., Jackson K.W., McNicholas E., Kawamura M.J., Abdallah W.M., Wicha M.S. (2004). Role of Notch signaling in cell-fate determination of human mammary stem/progenitor cells. Breast Cancer Res..

[200] Bouras T., Pal B., Vaillant F., Harburg G., Asselin-Labat M.-L., Oakes S.R., Lindeman G.J., Visvader J.E. (2008). Notch signaling regulates mammary stem cell function and luminal cell-fate commitment. Cell Stem Cell.

[201] Grudzien P., Lo S., Albain K.S., Robinson P., Rajan P., Strack P.R., Golde T.E., Miele L., Foreman K.E. (2010). Inhibition of Notch signaling reduces the stem-like population of breast cancer cells and prevents mammosphere formation. Anticancer Res..

[202] Farnie G., Clarke R.B., Spence K., Pinnock N., Brennan K., Anderson N.G., Bundred N.J. (2007). Novel cell culture technique for primary ductal carcinoma in situ: Role of Notch and epidermal growth factor receptor signaling pathways. J. Natl. Cancer Inst..

[203] Harrison H., Farnie G., Howell S.J., Rock R.E., Stylianou S., Brennan K.R., Bundred N.J., Clarke R.B. (2010). Regulation of breast cancer stem cell activity by signaling through the Notch4 receptor. Cancer Res..

[204] Sansone P., Storci G., Tavolari S., Guarnieri T., Giovannini C., Taffurelli M., Ceccarelli C., Santini D., Paterini P., Marcu K.B. (2007). IL-6 triggers malignant features in mammospheres from human ductal breast carcinoma and normal mammary gland. J. Clin. Investig..

[205] Sansone P., Storci G., Giovannini C., Pandolfi S., Pianetti S., Taffurelli M., Santini D., Ceccarelli C., Chieco P., Bonafe M. (2007). p66Shc/Notch-3 interplay controls self-renewal and hypoxia survival in human stem/progenitor cells of the mammary gland expanded in vitro as mammospheres. Stem Cells.

[206] Zhao D., Mo Y., Li M.-T., Zou S.-W., Cheng Z.-L., Sun Y.-P., Xiong Y., Guan K.-L., Lei Q.-Y. (2014). NOTCH-induced aldehyde dehydrogenase 1A1 deacetylation promotes breast cancer stem cells. J. Clin. Investig..

[207] Mamaeva V., Niemi R., Beck M., Ozliseli E., Desai D., Landor S., Gronroos T., Kronqvist P., Pettersen I.K.N., McCormack E. (2016). Inhibiting Notch activity in breast cancer stem cells by glucose functionalized nanoparticles carrying gamma-secretase inhibitors. Mol. Ther..

[208] McGowan P.M., Simedrea C., Ribot E.J., Foster P.J., Palmieri D., Steeg P.S., Allan A.L., Chambers A.F. (2011). Notch1 inhibition alters the CD44hi/CD24lo population and reduces the formation of brain metastases from breast cancer. Mol. Cancer Res..

[209] Phillips T.M., McBride W.H., Pajonk F. (2006). The response of CD24(-/low)/CD44+ breast cancer-initiating cells to radiation. J. Natl. Cancer Inst..

[210] Kim R.-K., Kaushik N., Suh Y., Yoo K.-C., Cui Y.-H., Kim M.-J., Lee H.-J., Kim I.-G., Lee S.-J. (2016). Radiation driven epithelial-mesenchymal transition is mediated by Notch signaling in breast cancer. Oncotarget.

[211] Azzam D.J., Zhao D., Sun J., Minn A.J., Ranganathan P., Drews-Elger K., Han X., Picon-Ruiz M., Gilbert C.A., Wander S.A. (2013). Triple negative breast cancer initiating cell subsets differ in functional and molecular characteristics and in γ-secretase inhibitor drug responses. EMBO Mol. Med..

[212] Noseda M., McLean G., Niessen K., Chang L., Pollet I., Montpetit R., Shahidi R., Dorovini-Zis K., Li L., Beckstead B. (2004). Notch activation results in phenotypic and functional changes consistent with endothelial-to-mesenchymal transformation. Circ. Res..

[213] Timmerman L.A., Grego-Bessa J., Raya A., Bertran E., Perez-Pomares J.M., Diez J., Aranda S., Palomo S., McCormick F., Izpisua-Belmonte J.C. (2004). Notch promotes epithelial-mesenchymal transition during cardiac development and oncogenic transformation. Genes Dev..

[214] Niessen K., Fu Y., Chang L., Hoodless P.A., McFadden D., Karsan A. (2008). Slug is a direct Notch target required for initiation of cardiac cushion cellularization. J. Cell Biol..

[215] Leong K.G., Niessen K., Kulic I., Raouf A., Eaves C., Pollet I., Karsan A. (2007). Jagged1-mediated Notch activation induces epithelial-to-mesenchymal transition through Slug-induced repression of E-cadherin. J. Exp. Med..

[216] Shao S., Zhao X., Zhang X., Luo M., Zuo X., Huang S., Wang Y., Gu S., Zhao X. (2015). Notch1 signaling regulates the epithelial-mesenchymal transition and invasion of breast cancer in a Slug-dependent manner. Mol. Cancer.

[217] Sahlgren C., Gustafsson M.V., Jin S., Poellinger L., Lendahl U. (2008). Notch signaling mediates hypoxia-induced tumor cell migration and invasion. Proc. Natl. Acad. Sci. USA.

[218] Mittal S., Subramanyam D., Dey D., Kumar R.V., Rangarajan A. (2009). Cooperation of Notch and Ras/MAPK signaling pathways in human breast carcinogenesis. Mol. Cancer.

[219] Majumder M., Xin X., Liu L., Tutunea-Fatan E., Rodriguez-Torres M., Vincent K., Postovit L.-M., Hess D., Lala P.K. (2016). COX-2 induces breast cancer stem cells via EP4/PI3K/AKT/NOTCH/WNT axis. Stem Cells.

[220] Osipo C., Patel P., Rizzo P., Clementz A.G., Hao L., Golde T.E., Miele L. (2008). ErbB-2 inhibition activates Notch-1 and sensitizes breast cancer cells to a gamma-secretase inhibitor. Oncogene.

[221] Ju J., Yang W., Oh S., Nam K., Lee K., Noh D., Shin I. (2013). HER2 stabilizes survivin while concomitantly down-regulating survivin gene transcription by suppressing Notch cleavage. Biochem. J..

[222] Pandya K., Wyatt D., Gallagher B., Shah D., Baker A., Bloodworth J., Zlobin A., Pannuti A., Green A., Ellis I.O. (2016). PKCα attenuates Jagged-1-mediated Notch signaling in ErbB-2 positive breast cancer to reverse trastuzumab resistance. Clin. Cancer Res..

[223] Pandya K., Meeke K., Clementz A.G., Rogowski A., Roberts J., Miele L., Albain K.S., Osipo C. (2011). Targeting both Notch and ErbB-2 signalling pathways is required for prevention of ErbB-2-positive breast tumour recurrence. Br. J. Cancer.

[224] Rattner A., Hsieh J.C., Smallwood P.M., Gilbert D.J., Copeland N.G., Jenkins N.A., Nathans J. (1997). A family of secreted proteins contains homology to the cysteine-rich ligand-binding domain of frizzled receptors. Proc. Natl. Acad. Sci. USA.

[225] Hsieh J.-C., Lee L., Zhang L., Wefer S., Brown K., DeRossi C., Wines M.E., Rosenquist T., Holdener B.C. (2003). Mesd encodes an LRP5/6 chaperone essential for specification of mouse embryonic polarity. Cell.

[226] Mikels A.J., Nusse R. (2006). Wnts as ligands: Processing, secretion and reception. Oncogene.

[227] Tamai K., Zeng X., Liu C., Zhang X., Harada Y., Chang Z., He X. (2004). A mechanism for Wnt coreceptor activation. Mol. Cell.

[228] Behrens J., Jerchow B.A., Wurtele M., Grimm J., Asbrand C., Wirtz R., Kuhl M., Wedlich D., Birchmeier W. (1998). Functional interaction of an axin homolog, conductin, with beta-catenin, APC, and GSK3beta. Science.

[229] Seeling J.M., Miller J.R., Gil R., Moon R.T., White R., Virshup D.M. (1999). Regulation of beta-catenin signaling by the B56 subunit of protein phosphatase 2A. Science.

[230] Gao Z.-H., Seeling J.M., Hill V., Yochum A., Virshup D.M. (2002). Casein kinase I phosphorylates and destabilizes the beta-catenin degradation complex. Proc. Natl. Acad. Sci. USA.

[231] Mao J., Wang J., Liu B., Pan W., Farr G.H., Flynn C., Yuan H., Takada S., Kimelman D., Li L. (2001). Low-density lipoprotein receptor-related protein-5 binds to Axin and regulates the canonical Wnt signaling pathway. Mol. Cell.

[232] Brannon M., Gomperts M., Sumoy L., Moon R.T., Kimelman D. (1997). A beta-catenin/XTcf-3 complex binds to the siamois promoter to regulate dorsal axis specification in Xenopus. Genes Dev..

[233] Brunner E., Peter O., Schweizer L., Basler K. (1997). pangolin encodes a Lef-1 homologue that acts downstream of Armadillo to transduce the Wingless signal in Drosophila. Nature.

[234] Chu E.Y., Hens J., Andl T., Kairo A., Yamaguchi T.P., Brisken C., Glick A., Wysolmerski J.J., Millar S.E. (2004). Canonical WNT signaling promotes mammary placode development and is essential for initiation of mammary gland morphogenesis. Development.

[235] Veltmaat J.M., Van Veelen W., Thiery J.P., Bellusci S. (2004). Identification of the mammary line in mouse by Wnt10b expression. Dev. Dyn..

[236] Van Amerongen R., Bowman A.N., Nusse R. (2012). Developmental stage and time dictate the fate of Wnt/beta-catenin-responsive stem cells in the mammary gland. Cell Stem Cell.

[237] Lamb R., Ablett M.P., Spence K., Landberg G., Sims A.H., Clarke R.B. (2013). Wnt pathway activity in breast cancer sub-types and stem-like cells. PLoS ONE.

[238] Khalil S., Tan G.A., Giri D.D., Zhou X.K., Howe L.R. (2012). Activation status of Wnt/ss-catenin signaling in normal and neoplastic breast tissues: Relationship to HER2/neu expression in human and mouse. PLoS ONE.

[239] Woodward W.A., Chen M.S., Behbod F., Alfaro M.P., Buchholz T.A., Rosen J.M. (2007). WNT/β-catenin mediates radiation resistance of mouse mammary progenitor cells. Proc. Natl. Acad. Sci. USA.

[240] Wu Y., Ginther C., Kim J., Mosher N., Chung S., Slamon D., Vadgama J.V. (2012). Expression of Wnt3 activates Wnt/beta-catenin pathway and promotes EMT-like phenotype in trastuzumab-resistant HER2-overexpressing breast cancer cells. Mol. Cancer Res..

[241] Eger A., Stockinger A., Schaffhauser B., Beug H., Foisner R. (2000). Epithelial mesenchymal transition by c-Fos estrogen receptor activation involves nuclear translocation of beta-catenin and upregulation of beta-catenin/lymphoid enhancer binding factor-1 transcriptional activity. J. Cell Biol..

[242] Kim K., Lu Z., Hay E.D. (2002). Direct evidence for a role of beta-catenin/LEF-1 signaling pathway in induction of EMT. Cell Biol. Int..

[243] Plaks V., Brenot A., Lawson D.A., Linneman J., Van Kappel E.C., Wong K., de Sauvage F., Klein O.D., Werb Z. (2013). Lgr5 expressing cells are sufficient and necessary for postnatal mammary gland organogenesis. Cell Rep..

[244] Oskarsson T., Acharyya S., Zhang X.H.-F., Vanharanta S., Tavazoie S.F., Morris P.G., Downey R.J., Manova-Todorova K., Brogi E., Massagué J. (2011). Breast cancer cells produce tenascin C as a metastatic niche component to colonize the lungs. Nat. Med..

[245] Yang L., Tang H., Kong Y., Xie X., Chen J., Song C., Liu X., Ye F., Li N., Wang N. (2015). LGR5 promotes breast cancer progression and maintains stem-like cells through activation of Wnt/beta-catenin signaling. Stem Cells.

[246] Chang Y.-W., Su Y.-J., Hsiao M., Wei K.-C., Lin W.-H., Liang C.-L., Chen S.-C., Lee J.-L. (2015). Diverse targets of beta-catenin during the epithelial-mesenchymal transition define cancer stem cells and predict disease relapse. Cancer Res..

[247] Schroeder J.A., Adriance M.C., McConnell E.J., Thompson M.C., Pockaj B., Gendler S.J. (2002). ErbB-beta-catenin complexes are associated with human infiltrating ductal breast and murine mammary tumor virus (MMTV)-Wnt-1 and MMTV-c-Neu transgenic carcinomas. J. Biol. Chem..

[248] Wang K., Ma Q., Ren Y., He J., Zhang Y., Zhang Y., Chen W. (2007). Geldanamycin destabilizes HER2 tyrosine kinase and suppresses Wnt/beta-catenin signaling in HER2 overexpressing human breast cancer cells. Oncol. Rep..

[249] Yamaguchi H., Chang S.-S., Hsu J.L., Hung M.-C. (2014). Signaling cross-talk in the resistance to HER family receptor targeted therapy. Oncogene.

[250] Khramtsov A.I., Khramtsova G.F., Tretiakova M., Huo D., Olopade O.I., Goss K.H. (2010). Wnt/β-catenin pathway activation is enriched in basal-like breast cancers and predicts poor outcome. Am. J. Pathol..

[251] Dey N., Young B., Abramovitz M., Bouzyk M., Barwick B., De P., Leyland-Jones B. (2013). Differential activation of Wnt-β-catenin pathway in triple negative breast cancer increases MMP7 in a PTEN dependent manner. PLoS ONE.

[252] Stahl N., Boulton T.G., Farruggella T., Ip N.Y., Davis S., Witthuhn B.A., Quelle F.W., Silvennoinen O., Barbieri G., Pellegrini S. (1994). Association and activation of Jak-Tyk kinases by CNTF-LIF-OSM-IL-6 beta receptor components. Science.

[253] Argetsinger L.S., Campbell G.S., Yang X., Witthuhn B.A., Silvennoinen O., Ihle J.N., Carter-Su C. (1993). Identification of JAK2 as a growth hormone receptor-associated tyrosine kinase. Cell.

[254] Heim M.H., Kerr I.M., Stark G.R., Darnell J.E.J. (1995). Contribution of STAT SH2 groups to specific interferon signaling by the Jak-STAT pathway. Science.

[255] Shuai K., Ziemiecki A., Wilks A.F., Harpur A.G., Sadowski H.B., Gilman M.Z., Darnell J.E. (1993). Polypeptide signalling to the nucleus through tyrosine phosphorylation of Jak and Stat proteins. Nature.

[256] Vinkemeier U., Cohen S.L., Moarefi I., Chait B.T., Kuriyan J., Darnell J.E.J. (1996). DNA binding of in vitro activated Stat1 alpha, Stat1 beta and truncated Stat1: Interaction between NH2-terminal domains stabilizes binding of two dimers to tandem DNA sites. EMBO J..

[257] Wang X., Wang G., Zhao Y., Liu X., Ding Q., Shi J., Ding Y., Wang S. (2012). STAT3 mediates resistance of CD44(+)CD24(-/low) breast cancer stem cells to tamoxifen in vitro. J. Biomed. Res..

[258] Wei W., Tweardy D.J., Zhang M., Zhang X., Landua J., Petrovic I., Bu W., Roarty K., Hilsenbeck S.G., Rosen J.M. (2014). STAT3 signaling is activated preferentially in tumor-initiating cells in claudin-low models of human breast cancer. Stem Cells.

[259] Lin L., Hutzen B., Lee H.-F., Peng Z., Wang W., Zhao C., Lin H.-J., Sun D., Li P.-K., Li C. (2013). Evaluation of STAT3 signaling in ALDH+ and ALDH+/CD44+/CD24− subpopulations of breast cancer cells. PLoS ONE.

[260] Dave B., Landis M.D., Tweardy D.J., Chang J.C., Dobrolecki L.E., Wu M.-F., Zhang X., Westbrook T.F., Hilsenbeck S.G., Liu D. (2012). Selective small molecule Stat3 inhibitor reduces breast cancer tumor-initiating cells and improves recurrence free survival in a human-xenograft model. PLoS ONE.

[261] So J.Y., Smolarek A.K., Salerno D.M., Maehr H., Uskokovic M., Liu F., Suh N. (2013). Targeting CD44-STAT3 signaling by Gemini vitamin D analog leads to inhibition of invasion in basal-like breast cancer. PLoS ONE.

[262] Hernandez-Vargas H., Ouzounova M., Le Calvez-Kelm F., Lambert M.-P., McKay-Chopin S., Tavtigian S.V., Puisieux A., Matar C., Herceg Z. (2011). Methylome analysis reveals Jak-STAT pathway deregulation in putative breast cancer stem cells. Epigenetics.

[263] Sullivan N.J., Sasser A.K., Axel A.E., Vesuna F., Raman V., Ramirez N., Oberyszyn T.M., Hall B.M. (2009). Interleukin-6 induces an epithelial-mesenchymal transition phenotype in human breast cancer cells. Oncogene.

[264] Xie G., Yao Q., Liu Y., Du S., Liu A., Guo Z., Sun A., Ruan J., Chen L., Ye C., Yuan Y. (2012). IL-6-induced epithelial-mesenchymal transition promotes the generation of breast cancer stem-like cells analogous to mammosphere cultures. Int. J. Oncol..

[265] Zhang F., Li C., Halfter H., Liu J. (2003). Delineating an oncostatin M-activated STAT3 signaling pathway that coordinates the expression of genes involved in cell cycle regulation and extracellular matrix deposition of MCF-7 cells. Oncogene.

[266] Park J., Schwarzbauer J.E. (2014). Mammary epithelial cell interactions with fibronectin stimulate epithelial-mesenchymal transition. Oncogene.

[267] Guo L., Chen C., Shi M., Wang F., Chen X., Diao D., Hu M., Yu M., Qian L., Guo N. (2013). Stat3-coordinated Lin-28-let-7-HMGA2 and miR-200-ZEB1 circuits initiate and maintain oncostatin M-driven epithelial-mesenchymal transition. Oncogene.

[268] Davis F.M., Azimi I., Faville R.A., Peters A.A., Jalink K., Putney J.W.J., Goodhill G.J., Thompson E.W., Roberts-Thomson S.J., Monteith G.R. (2014). Induction of epithelial-mesenchymal transition (EMT) in breast cancer cells is calcium signal dependent. Oncogene.

[269] Olayioye M.A., Beuvink I., Horsch K., Daly J.M., Hynes N.E. (1999). ErbB receptor-induced activation of stat transcription factors is mediated by Src tyrosine kinases. J. Biol. Chem..

[270] DeArmond D., Brattain M.G., Jessup J.M., Kreisberg J., Malik S., Zhao S., Freeman J.W. (2003). Autocrine-mediated ErbB-2 kinase activation of STAT3 is required for growth factor independence of pancreatic cancer cell lines. Oncogene.

[271] Hawthorne V.S., Huang W.-C., Neal C.L., Tseng L.-M., Hung M.-C., Yu D. (2009). ErbB2-mediated Src and STAT3 activation leads to transcriptional ppregulation of p21(Cip1) and chemoresistance in breast cancer cells. Mol. Cancer Res..

[272] Barbieri I., Quaglino E., Maritano D., Pannellini T., Riera L., Cavallo F., Forni G., Musiani P., Chiarle R., Poli V. (2010). Stat3 is required for anchorage-independent growth and metastasis but not for mammary tumor development downstream of the ErbB-2 oncogene. Mol. Carcinog..

[273] Hartman Z.C., Yang X.-Y., Glass O., Lei G., Osada T., Dave S.S., Morse M.A., Clay T.M., Lyerly H.K. (2011). HER2 overexpression elicits a pro-inflammatory IL-6 autocrine signaling loop that is critical for tumorigenesis. Cancer Res..

[274] Chung S.S., Giehl N., Wu Y., Vadgama J.V. (2014). STAT3 activation in HER2-overexpressing breast cancer promotes epithelial-mesenchymal transition and cancer stem cell traits. Int. J. Oncol..

[275] Espinoza I., Pochampally R., Xing F., Watabe K., Miele L. (2013). Notch signaling: Targeting cancer stem cells and epithelial-to-mesenchymal transition. Onco. Targets. Ther..

[276] Echelard Y., Epstein D.J., St-Jacques B., Shen L., Mohler J., McMahon J.A., McMahon A.P. (1993). Sonic hedgehog, a member of a family of putative signaling molecules, is implicated in the regulation of CNS polarity. Cell.

[277] Lanske B., Karaplis A.C., Lee K., Luz A., Vortkamp A., Pirro A., Karperien M., Defize L.H., Ho C., Mulligan R.C. (1996). PTH/PTHrP receptor in early development and Indian hedgehog-regulated bone growth. Science.

[278] Parmantier E., Lynn B., Lawson D., Turmaine M., Namini S.S., Chakrabarti L., McMahon A.P., Jessen K.R., Mirsky R. (1999). Schwann cell-derived Desert hedgehog controls the development of peripheral nerve sheaths. Neuron.

[279] Alcedo J., Ayzenzon M., Von Ohlen T., Noll M., Hooper J.E. (1996). The Drosophila smoothened gene encodes a seven-pass membrane protein, a putative receptor for the hedgehog signal. Cell.

[280] Corbit K.C., Aanstad P., Singla V., Norman A.R., Stainier D.Y.R., Reiter J.F. (2005). Vertebrate Smoothened functions at the primary cilium. Nature.

[281] Kinzler K.W., Vogelstein B. (1990). The GLI gene encodes a nuclear protein which binds specific sequences in the human genome. Mol. Cell. Biol..

[282] Kinzler K.W., Ruppert J.M., Bigner S.H., Vogelstein B. (1988). The GLI gene is a member of the Kruppel family of zinc finger proteins. Nature.

[283] Chen M.-H., Wilson C.W., Li Y.-J., Law K.K.L., Lu C.-S., Gacayan R., Zhang X., Hui C., Chuang P.-T. (2009). Cilium-independent regulation of Gli protein function by Sufu in Hedgehog signaling is evolutionarily conserved. Genes Dev..

[284] Cheung H.O.-L., Zhang X., Ribeiro A., Mo R., Makino S., Puviindran V., Law K.K.L., Briscoe J., Hui C.-C. (2009). The kinesin protein Kif7 is a critical regulator of Gli transcription factors in mammalian hedgehog signaling. Sci. Signal..

[285] Cochrane C.R., Szczepny A., Watkins D.N., Cain J.E. (2015). Hedgehog signaling in the maintenance of cancer stem cells. Cancers.

[286] Zhao H., Tang H., Xiao Q., He M., Zhao L., Fu Y., Wu H., Yu Z., Jiang Q., Yan Y. (2016). The Hedgehog signaling pathway is associated with poor prognosis in breast cancer patients with the CD44+/CD24 phenotype. Mol. Med. Rep..

[287] Shipitsin M., Campbell L.L., Argani P., Weremowicz S., Bloushtain-Qimron N., Yao J., Nikolskaya T., Serebryiskaya T., Beroukhim R., Hu M. (2007). Molecular definition of breast tumor heterogeneity. Cancer Cell.

[288] Liu S., Dontu G., Mantle I.D., Patel S., Ahn N., Jackson K.W., Suri P., Wicha M.S. (2006). Hedgehog signaling and Bmi-1 regulate self-renewal of normal and malignant human mammary stem cells. Cancer Res..

[289] Tanaka H., Nakamura M., Kameda C., Kubo M., Sato N., Kuroki S., Tanaka M., Katano M. (2009). The Hedgehog signaling pathway plays an essential role in maintaining the CD44+CD24-/low subpopulation and the side population of breast cancer cells. Anticancer Res..

[290] Yang N., Zhou T.-C., Lei X., Wang C., Yan M., Wang Z.-F., Liu W., Wang J., Ming K.-H., Wang B.-C. (2016). Inhibition of Sonic Hedgehog signaling pathway by thiazole antibiotic thiostrepton attenuates the CD44+/CD24− stem-like population and sphere-forming capacity in triple-negative breast cancer. Cell. Physiol. Biochem..

[291] He M., Fu Y., Yan Y., Xiao Q., Wu H., Yao W., Zhao H., Zhao L., Jiang Q., Yu Z. (2015). The Hedgehog signalling pathway mediates drug response of MCF-7 mammosphere cells in breast cancer patients. Clin. Sci..

[292] Sun M., Zhang N., Wang X., Li Y., Qi W., Zhang H., Li Z., Yang Q. (2016). Hedgehog pathway is involved in nitidine chloride induced inhibition of epithelial-mesenchymal transition and cancer stem cells-like properties in breast cancer cells. Cell Biosci..

[293] Lu Y., Ma W., Mao J., Yu X., Hou Z., Fan S., Song B., Wang H., Li J., Kang L. (2015). Salinomycin exerts anticancer effects on human breast carcinoma MCF-7 cancer stem cells via modulation of Hedgehog signaling. Chem. Biol. Interact..

[294] Fu Y.-Z., Yan Y.-Y., He M., Xiao Q.-H., Yao W.-F., Zhao L., Wu H.-Z., Yu Z.-J., Zhou M.-Y., Lv M.-T. (2016). Salinomycin induces selective cytotoxicity to MCF-7 mammosphere cells through targeting the Hedgehog signaling pathway. Oncol. Rep..

[295] Memmi E.M., Sanarico A.G., Giacobbe A., Peschiaroli A., Frezza V., Cicalese A., Pisati F., Tosoni D., Zhou H., Tonon G. (2015). p63 Sustains self-renewal of mammary cancer stem cells through regulation of Sonic Hedgehog signaling. Proc. Natl. Acad. Sci. USA.

[296] Han B., Qu Y., Yu-Rice Y., Johnson J., Cui X. (2016). FOXC1-induced Gli2 activation: A non-canonical pathway contributing to stemness and anti-Hedgehog resistance in basal-like breast cancer. Mol. Cell. Oncol..

[297] Benvenuto M., Masuelli L., De Smaele E., Fantini M., Mattera R., Cucchi D., Bonanno E., Di Stefano E., Frajese G.V., Orlandi A. (2016). In vitro and in vivo inhibition of breast cancer cell growth by targeting the Hedgehog/GLI pathway with SMO (GDC-0449) or GLI (GANT-61) inhibitors. Oncotarget.

[298] Matevossian A., Resh M.D. (2015). Hedgehog Acyltransferase as a target in estrogen receptor positive, HER2 amplified, and tamoxifen resistant breast cancer cells. Mol. Cancer.

[299] Ramaswamy B., Lu Y., Teng K., Nuova G., Li X., Shapiro C.L., Majumder S. (2012). Hedgehog signaling is a novel therapeutic target in tamoxifen-resistant breast cancer aberrantly activated by PI3K/AKT pathway. Cancer Res..

[300] Kern D., Regl G., Hofbauer S.W., Altenhofer P., Achatz G., Dlugosz A., Schnidar H., Greil R., Hartmann T.N., Aberger F. (2015). Hedgehog/GLI and PI3K signaling in the initiation and maintenance of chronic lymphocytic leukemia. Oncogene.

[301] Ke Z., Caiping S., Qing Z., Xiaojing W. (2015). Sonic hedgehog-Gli1 signals promote epithelial-mesenchymal transition in ovarian cancer by mediating PI3K/AKT pathway. Med. Oncol..

[302] Sharma N., Nanta R., Sharma J., Gunewardena S., Singh K.P., Shankar S., Srivastava R.K. (2015). PI3K/AKT/mTOR and sonic hedgehog pathways cooperate together to inhibit human pancreatic cancer stem cell characteristics and tumor growth. Oncotarget.

[303] Kebenko M., Drenckhan A., Gros S.J., Jucker M., Grabinski N., Ewald F., Grottke A., Schultze A., Izbicki J.R., Bokemeyer C. (2015). ErbB2 signaling activates the Hedgehog pathway via PI3K-Akt in human esophageal adenocarcinoma: Identification of novel targets for concerted therapy concepts. Cell. Signal..

[304] Radisky E.S., Radisky D.C. (2015). Matrix metalloproteinases as breast cancer drivers and therapeutic targets. Front. Biosci..

[305] Cathcarta J., Pulkoski-Grossa A., Cao J. (2015). Targeting matrix metalloproteinases in cancer: Bringing new life to old ideas. Genes Dis..

